# Diverged Alleles of the *Anopheles gambiae* Leucine-Rich Repeat Gene *APL1A* Display Distinct Protective Profiles against *Plasmodium falciparum*

**DOI:** 10.1371/journal.pone.0052684

**Published:** 2012-12-28

**Authors:** Inge Holm, Catherine Lavazec, Thierry Garnier, Christian Mitri, Michelle M. Riehle, Emmanuel Bischoff, Emma Brito-Fravallo, Eizo Takashima, Isabelle Thiery, Agnes Zettor, Stephane Petres, Catherine Bourgouin, Kenneth D. Vernick, Karin Eiglmeier

**Affiliations:** 1 Unit of Insect Vector Genetics and Genomics, Department of Parasitology and Mycology, CNRS Unit URA3012: Hosts, Vectors and Infectious Agents, Institut Pasteur, Paris, France; 2 Department of Microbiology, University of Minnesota, Saint Paul, Minnesota, United States of America; 3 Centre de Production et Infection des Anophèles (CEPIA), Institut Pasteur, Paris, France; 4 Centre de Production de Protéines recombinantes et d’Anticorps, Institut Pasteur, Paris, France; Kansas State University, United States of America

## Abstract

Functional studies have demonstrated a role for the *Anopheles gambiae APL1A* gene in resistance against the human malaria parasite, *Plasmodium falciparum*. Here, we exhaustively characterize the structure of the *APL1* locus and show that three structurally different *APL1A* alleles segregate in the Ngousso colony. Genetic association combined with RNAi-mediated gene silencing revealed that *APL1A* alleles display distinct protective profiles against *P. falciparum*. One *APL1A* allele is sufficient to explain the protective phenotype of *APL1A* observed in silencing experiments. Epitope-tagged APL1A isoforms expressed in an *in vitro* hemocyte-like cell system showed that under assay conditions, the most protective APL1A isoform (APL1A^2^) localizes within large cytoplasmic vesicles, is not constitutively secreted, and forms only one protein complex, while a less protective isoform (APL1A^1^) is constitutively secreted in at least two protein complexes. The tested alleles are identical to natural variants in the wild *A. gambiae* population, suggesting that *APL1A* genetic variation could be a factor underlying natural heterogeneity of vector susceptibility to *P. falciparum*.

## Introduction

Most of the global transmission of human malaria due to *Plasmodium falciparum* occurs in Sub-Saharan Africa and is vectored primarily by the mosquito *Anopheles gambiae* [1]. Studies on the *A. gambiae-P. falciparum* pair have revealed a large genetic influence upon mosquito resistance to the human malaria parasite [2]–[5], although the causative variants underlying the mapped quantitative trait loci (QTLs) have not yet been resolved. Separately, a number of functional studies show that the immune system of *A. gambiae* protects mosquitoes against infection with *P. falciparum*, and multiple immune factors have been identified [6]–[14].

In *A. gambiae* mosquitoes from West and East Africa, genetic loci strongly linked to *P. falciparum* resistance traits were repeatedly mapped to the same segment of chromosome 2L, and were resolved to a ∼10 Mb genetic interval [2], [4], [5]. The *APL1* (*Anopheles Plasmodium*-responsive leucine-rich repeat 1) genes were identified as candidates within the resistance QTL cluster. The *APL1* locus is ∼18 kilobases (kb) in length and comprises three paralogs, *APL1A*, *APL1B* and *APL1C* [15].

The three *APL1* genes encode a family of leucine-rich repeat (LRR) proteins that influence the survival of *Plasmodium* parasites, displaying distinct activities depending on the parasite species: APL1A protects against the human malaria parasite *P. falciparum* that is naturally transmitted by this vector, and APL1C against rodent malaria parasites [11], [15]. The APL1C protein has been shown to form a heterodimer with another LRR protein, LRIM1, as part of a functional complex with the complement C3-like protein TEP1 [16]–[19]. Protein partners of APL1A and APL1B remain unknown.

The *APL1* genes show a high degree of similarity in sequence and intron-exon structure, and they likely originate from gene duplication and diversification events. Each of the encoded APL1 proteins is characterized by the presence of a N-terminal signal sequence and a LRR region followed by a cysteine-rich tract, and some variants have a C-terminal coiled-coil domain. The *APL1* locus comprises two additional genes also coding for LRR proteins, *AGAP007034* (LRIM11) and *AGAP007037* (LRIM3), which are not members of the *APL1* family. Despite the overall structural resemblance with the APL1 proteins, these genes encode proteins belonging to the LRIM family [15], [19], [20].

In nature, *APL1* genes display high genetic diversity, in a pattern consistent with adaptive maintenance of polymorphism [21]. The differential functional roles, if any, of *APL1A* alleles are not known. However, the mapped *P. falciparum* resistance QTLs that include the *APL1* locus exert a strong influence on infection outcome [2], [4]. The genetically mapped phenotypic effect is due to as yet unknown causative allelic variant(s) found within the QTL locus that includes *APL1A*, which remains a strong candidate gene. One previous report described fine genetic dissection of an *A. gambiae* locus, but the locus was linked to protection against the rodent parasite *P. berghei*, and does not correspond to the location of a *P. falciparum*-protective QTL [22].

Here we measured the individual phenotypes of *APL1A* alleles for protection against *P. falciparum*, using the Ngousso laboratory colony, after first validating the existence of the same alleles in the natural population. By genetic association studies using RNA interference-mediated knockdown assays, we evaluated the importance of the structurally different allelic variants of the *APL1A* gene against the human malaria parasite *P. falciparum* and observed distinct allele-specific protective profiles. Finally, in assays using cultured hemocyte-like cells we detected different subcellular localization and secretion patterns of the APL1A allelic isoforms.

## Results

### Structure of the *APL1* Locus

**Figure 1:**
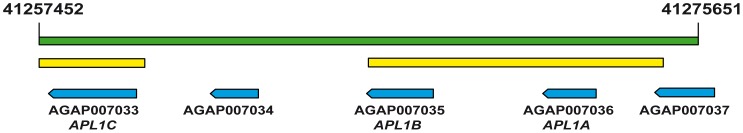
Genomic organisation of the *A. gambiae APL1* region. The genomic region of the 2L chromosome that comprises the *APL1* locus is indicated by a green bar (*A. gambiae* reference genome [23], genome assembly AgamP3). The bar delimits the region of the genome that has been completely spanned with overlapping PCR amplicons. The chromosomal positions are indicated (in bp) on top of the bar. The yellow bars indicate the regions for which overlapping PCR fragments have been sequenced. Blue arrows represent the genes in this chromosomal region and their direction of transcription. The last two lines indicate gene identifiers and names.

We generated overlapping PCR fragments over ∼18 kb covering the *APL1* locus from 20 individual Ngousso mosquitoes. The sizes of the obtained amplicons corresponded to the physical distances displayed in the *A. gambiae* PEST-strain reference genome [23], with the exception of fragments from two intergenic regions, the regions between *APL1B* - *APL1A* and *APL1A* – *AGAP007037*. These size differences are due to the presence of insertion-deletion variations (indels) and to the absence in Ngousso of the TA-III-Ag miniature inverted transposon (MITE transposable element) identified in PEST upstream of *APL1A* [15]. These results confirm that the *APL1* locus in Ngousso has the same genomic organization as the PEST reference, and did not reveal the presence of any additional copies of the *APL1* genes (Figure 1).

### Three *APL1A* Alleles Segregate in the Ngousso Colony

**Figure 2:**
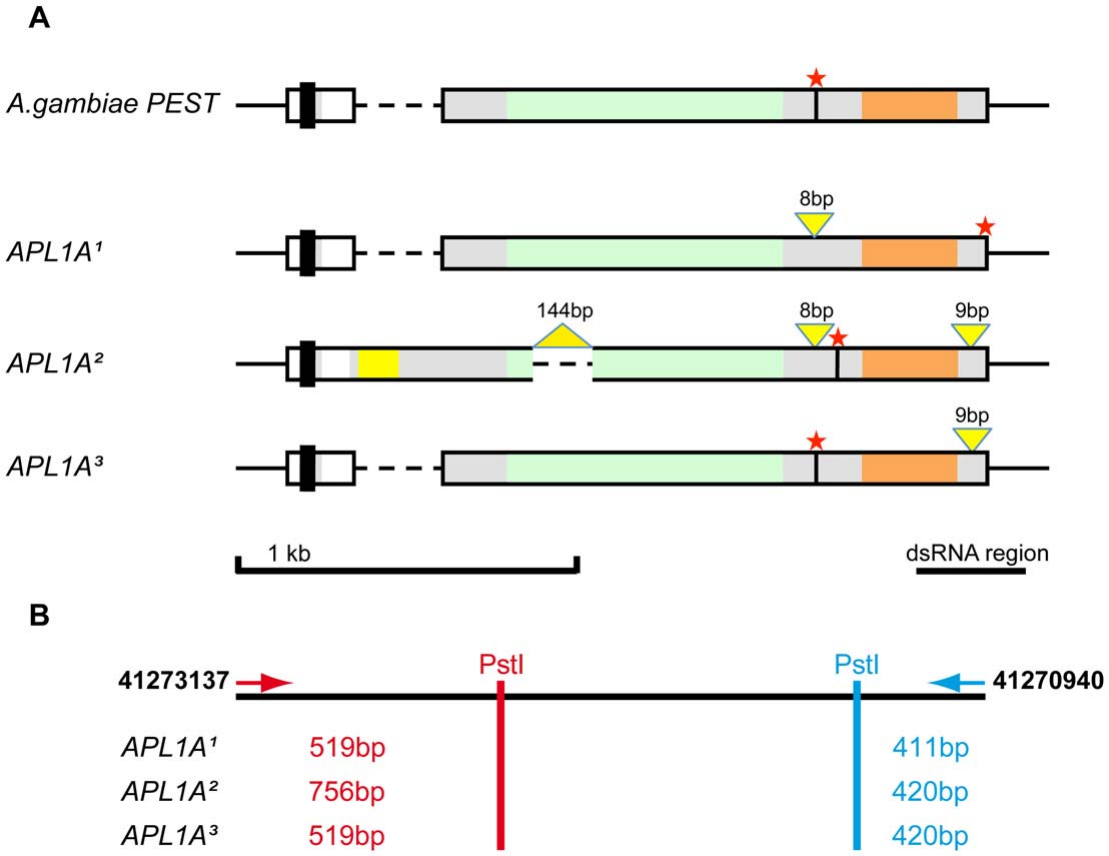
Alignment and comparison of the Ngousso *APL1A* alleles. A) Schematic view of the genomic regions of the different *APL1A* alleles identified in *A. gambiae* Ngousso. Each row represents the genomic region of one *APL1A* allele. The first row shows the *APL1A* gene from the *A. gambiae* reference genome. Each of the following three rows represents one of the *APL1A* alleles present in the Ngousso strain, *APL1A^1^*, *APL1A^2^*, *APL1A^3^,* respectively. The sequences belonging to the *APL1A* genes are represented as solid bars with white boxes corresponding to the 5′ non-coding region and the region of the first intron. Dashed lines illustrate introduced gaps for a better alignment of the different alleles. DNA regions adjacent of the *APL1A* genes are shown as black horizontal lines. Predicted peptide domains are indicated as follows: signal peptide (black vertical bar), additional sequences and PANGGL repeat region (yellow), LRR repeat region (green), coiled-coil domain (orange). Yellow triangles indicate the positions and length of indels, red stars indicate the positions of the translation stop codons. At the bottom right, the black bar indicates the DNA region targeted by the dsRNA. **B)** Schematic representation of the labelled *Pst*I-RFLP fragments of *APL1A* alleles obtained by the high-throughput RFLP genotyping assay in Ngousso. The first line represents the genomic DNA of this region with two arrows marking the positions of the forward and reverse fluorescent-labeled primers used in the assay. The red and the blue vertical bars indicate the respective position of the terminal *Pst*I restriction sites in the different alleles. The fragment sizes of the labelled *Pst*I fragment generated from the 5′ end of the Ngousso *APL1A* alleles are indicated in red. The sizes of the labelled *Pst*I fragment from the 3′ end of the *APL1A* alleles are in blue.

Each of the three *APL1* genes was amplified with specific primers flanking the coding region to avoid cross-amplification of the other paralogs due to their high similarity (Table S1). Sequencing of the diploid PCR fragments indicated a maximum of two alleles in each PCR reaction. We chose two homozygotes per allele and sequenced the cognate coding region. The structurally most polymorphic of the paralogs is *APL1A*, with three alleles: *APL1A^1^*, *APL1A^2^* and *APL1A^3^* (Figure 2, and Supplementary Information S1), following the previous nomenclature of gene name with allele superscript [15], [21]. All three Ngousso *APL1A* alleles present a similar overall structure, but show diagnostic indel polymorphisms (Figure S1). The diagnostic differences allowed us to develop a genotyping assay for the *APL1A* alleles. The *APL1A^3^* allele is structurally nearly identical to the *APL1A* of the PEST reference.

**Figure 3:**
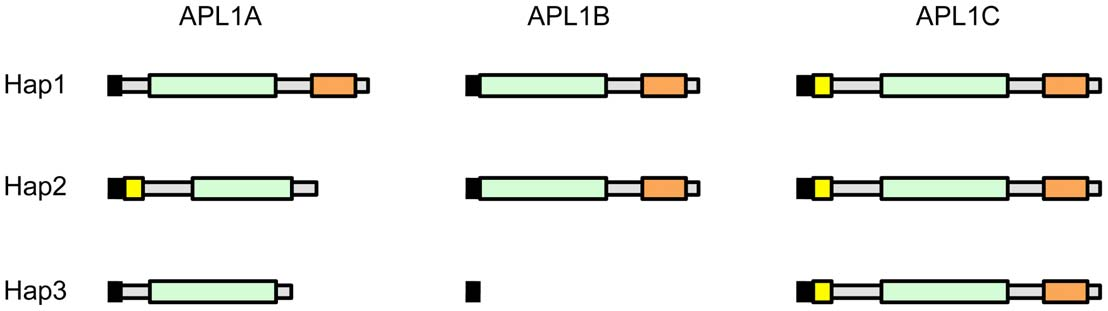
Schematic representation of the three different *APL1* haplotypes found in *A. gambiae* Ngousso. Each row shows one of the three identified *APL1* haplotypes of Ngousso, numbered 1 to 3. The three APL1 proteins encoded by each haplotype are shown. Predicted peptide domains are indicated as follows: black vertical bar – signal sequence, yellow box – PANGGL repeat region, green – LRR repeat region, orange – coiled-coil domain.

The presence of allele specific differences in *APL1A^2^* and *APL1A^3^* leads to premature stop codons that result in proteins without the coiled-coil domain (Figure 3, and Supplementary Information S1). The alleles, *APL1A^1^* and *APL1A^2^*, exist also in the G3 colony and in field caught mosquitoes from Mali [15], [21]. In addition, we found four *APL1A* alleles in a sample set of wild mosquitoes carrying the standard chromosomal form 2La^+^/2La^+^ from Burkina Faso (manuscript accepted [24]) and three of these alleles were structurally identical to Ngousso alleles *APL1A^1^, APL1A^2^* and *APL1A^3^,* overall indicating that the *APL1A* alleles observed in Ngousso are not colony artifacts or geographically limited forms. Instead, there appears to be a limited repertoire of allelic forms with major structural consequences for the encoded APL1A proteins.

### Alleles of *APL1B* and *APL1C* in Ngousso and Haplotype Organization of the *APL1* Locus

Three *APL1B* allelic variants exist in Ngousso. The *APL1B^1^* and *APL1B^2^* alleles display only single nucleotide polymorphism (SNP) differences, and the encoded proteins correspond to the APL1B protein in PEST. The *APL1B^3^* allele, however, has a premature stop codon due to a point mutation at the beginning of the second exon (2L: 41268220), resulting in a 21 amino acid product comprising essentially just the signal peptide. We confirmed the existence of this truncated coding sequence variant in genome sequences from multiple samples from the Ngousso colony.

For the *APL1C* gene, the major allelic differences are due to variations in the copy number and composition of the encoded consensus amino acid sequence Pro-Ala-Asn-Gly-Gly-Leu (PANGGL) repeat in the N-terminal region of APL1C. The PANGGL repeat or its variants is encoded by all *APL1C* alleles and is also present in APL1A^2^ but absent in the other APL1A alleles and all APL1B alleles. This amino acid repeat motif is not found in any other *Anopheles* genes, and its function is unknown.

In order to detect linkage between alleles, we sequenced the complete coding sequence of *APL1B* and *APL1C* from two mosquitoes homozygous for each of the three *APL1A* alleles (n = 6 for complete locus sequence). Alleles of the paralogs are linked to form three stable haplotypes in Ngousso (Figure 3). Thus, each *APL1A* allele is consistently part of a specific combination of *APL1B* and *C* alleles. For example, the *APL1A^3^*-bearing haplotype also encodes the highly truncated APL1B^3^ protein along with a complete APL1C protein. The haplotype structure of the locus in wild mosquitoes is unknown, but the founding effect of colonization should in general produce more extensive haplotype structure than exists in nature.

### The *APL1A^2^* Allele is Highly Protective against *P. falciparum* Infection

We previously showed that *APL1A* displays a protective function against the human malaria parasite *P. falciparum* in *A. gambiae* Ngousso using gene silencing experiments [11]. Here, we examined the relative contribution of the three *APL1A* alleles to host protection. The high sequence similarity between the three *APL1A* alleles does not permit the design of double-stranded RNAs (dsRNAs) for allele-specific knockdown. Consequently, for gene silencing we injected a single dsRNA targeting all three *APL1A* alleles (Figure S5) followed by infection challenge and individual mosquito genotyping to test association of infection status with *APL1A* allele. We used gene silencing of *APL1A* to specifically measure the APL1A effect, and isolate it from the influence, if any, of the linked alleles on the *APL1*-bearing haplotype. The haplotype is of unknown length, and could include the effects, possibly even contradictory in terms of phenotype, of tens or more linked alleles, including but not limited to *APL1B* and *APL1C*.

**Figure 4:**
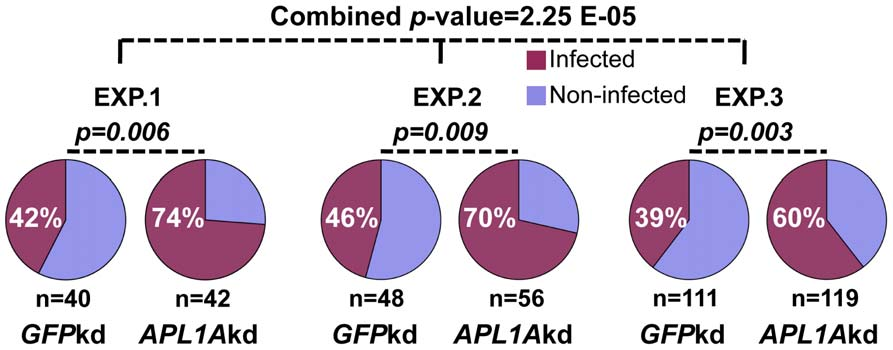
*APL1A* silencing renders mosquitoes significantly more susceptible to *P. falciparum* infection. Silencing of *APL1A* in *A. gambiae* Ngousso results in significantly higher infection prevalence in mosquitoes fed on *in vitro* cultured *P. falciparum* gametocytes. For each experiment (EXP.), a Chi-square analysis was performed to compare infection prevalence between *GFP-*knockdown (*GFP*kd) and *APL1A*kd. A meta-analysis using the Fisher method [33] was also used to combine the *p*-values of the three independent experiments.

We compared the level of *P. falciparum* infection for each *APL1A* allele between the states of normal APL1A function (mosquitoes treated with control dsRNA ds*GFP*) and APL1A loss-of-function (treated with dsRNA for *APL1A*, ds*APL1A*). In each of three independent replicate infections (Table S2A, Figure S2), APL1A was strongly protective, because its silencing rendered mosquitoes significantly more susceptible to *P. falciparum* infection by increasing infection prevalence (combined *p*-value for infection prevalence, 2.25e-05; Figure 4), but without affecting infection intensity (Figure S2).

When analyzing the results by *APL1A* allele, only the *APL1A^2^* allele displayed a consistent protective effect, because the global silencing of all *APL1A* alleles caused a highly significant increase of infection prevalence only among carriers of the *APL1A^2^* allele (*p* = 5.07e-07; Table 1). The protective phenotype is seen in *APL1A^2^* carriers regardless of the other *APL1A* allele present in the diploid mosquito, and thus demonstrates a genetically and functionally dominant protective effect of the *APL1A^2^* allele against *P. falciparum* infection. There was insufficient sample size to test whether the *APL1A^2^/APL1A^2^* homozygous genotype is even more protective than the *APL1A^2^* allele alone.

**Table 1:** *APL1A* alleles display distinct protective function against *P. falciparum* infection.

		*GFP*kd		*APL1A*kd			
*APL1A* alleles	EXP.	Inf. Prevalence	(n)	Inf. Prevalence	(n)	*p*-value (1)	Combined *p*-value (2)
*APL1A^1^*	EXP.1	59%	22	73%	26	0.368	0.00059
	EXP.2	41%	17	65%	23	0.194	
	EXP.3	28%	50	67%	46	0.0001	
*APL1A^2^*	EXP.1	37%	40	77%	35	0.0008	5.07E-07
	EXP.2	43%	54	74%	68	0.0006	
	EXP.3	44%	113	62%	119	0.0050	
*APL1A^3^*	EXP.1	33%	18	63%	19	0.104	0.24722
	EXP.2	60%	23	71%	21	0.535	
	EXP.3	43%	53	52%	63	0.351	

(1) Chi-square *p*-value comparing *GFP*kd *versus APL1A*kd for each experiment (EXP.).

(2) For each allele, the *p*-value of the 3 experiments were combined by meta-analysis.

This table presents the results from three independent *APL1A* gene-silencing assays (shown in Figure 4). From each mosquito both *APL1A* alleles were determined using the *APL1A*-RFLP test. For each gene-silencing treatment (*GFP*kd or *APL1A*kd) and for each experiment (EXP.), the oocyst infection prevalence (Inf. Prevalence) is given. The column labeled (n) indicates the sum of the corresponding *APL1A* allele in the mosquitoes of the experiment. Significance was calculated by Chi-square analysis, comparing the infection prevalence between *GFP*kd and *APL1A*kd mosquitoes for each experiment and for each allele (*APL1A^1^*, *APL1A^2^, APL1A^3^).* A meta-analysis using the Fisher method was also used to combine *p*-values of the three independent experiments.

No statistically significant allele specific effect on the infection intensities was detected in the mosquitoes of the three replicates injected with ds*APL1A* (data not shown).

In contrast, mosquitoes carrying the *APL1A^3^* allele showed no significant difference in infection outcome after ds*APL1A* treatment as compared to ds*GFP*-treated controls (*p* = 0.24722), indicating that function of the *APL1A^3^* allele does not underlie and is not required for the protection against *P. falciparum* mediated by the global silencing of *APL1A*. Finally, allele *APL1A^1^* carriers displayed a significant difference of infection in only one replicate. The combined *p*-value for *APL1A^1^* influence on infection prevalence is significant (*p* = 0.00059), although three orders of magnitude less than for *APL1A^2^*, but we regard it with caution because replicates 1 and 2, which were individually not significant, were still not significant when pooled to a single group before *p*-value calculation, indicating that the significant value in the *APL1A^1^* combined meta-analysis was generated solely by replicate 3. Statistical power analysis indicates that sample sizes were sufficient to detect an effect of *APL1^1^* and *APL1A^3^*, if one exists. For both *APL1A^1^* and *APL1A^3^*, there is power (>0.80 probability) to detect a difference in infection prevalence of the same magnitude as the effect of *APL1A^2^* (∼30% infection difference) in either replicate 3 alone, or by pooling replicates 1 and 2. Thus, the experiments were properly powered and the result for *APL1A^3^* can be considered a robust negative. For *APL1A^1^*, replicates 1 and 2 were robustly negative, but due to replicate 3 we regard *APL1A^1^* as at most weakly protective against *P. falciparum*. Taken together, these results show that, at least in the Ngousso colony, the *APL1A^2^* allele is necessary and sufficient to explain the protective effect of *APL1A* against *P. falciparum*, while *APL1A^3^* plays no part in protection, and *APL1A^1^* has no, or at most weak, effect.

### Role of Ngousso *APL1A* Haplotypes in *P. falciparum* Infections

We used the *APL1A* allele genotyping assay to test for an effect upon infection of the entire linked haplotype, rather than *APL1A* alone as tested in the dsRNA silencing assays. In three independent replicates (with n = 300 mosquitoes per replicate), mosquitoes were infected with *P. falciparum* in the absence of the dsRNA treatment used above (Table S2B, Figure S3). Despite the large sample sets, no significant association was observed between *APL1A* haplotypes as alleles or genotypes (data not shown). In each replicate, the observed genotype distribution was in Hardy-Weinberg equilibrium and each replicate had sufficient statistical power (>0.8 probability) to detect differences in infection prevalence of ≥20% between mosquitoes carrying different *APL1A* alleles, thus substantiating a robust negative result for haplotype effect. Sample sizes of haplotype combinations were too small (Figure S4) to test for genotype effects (data not shown).

### *APL1A* Alleles Encode Proteins with Different Subcellular Localization and Patterns of Secretion

**Figure 5:**
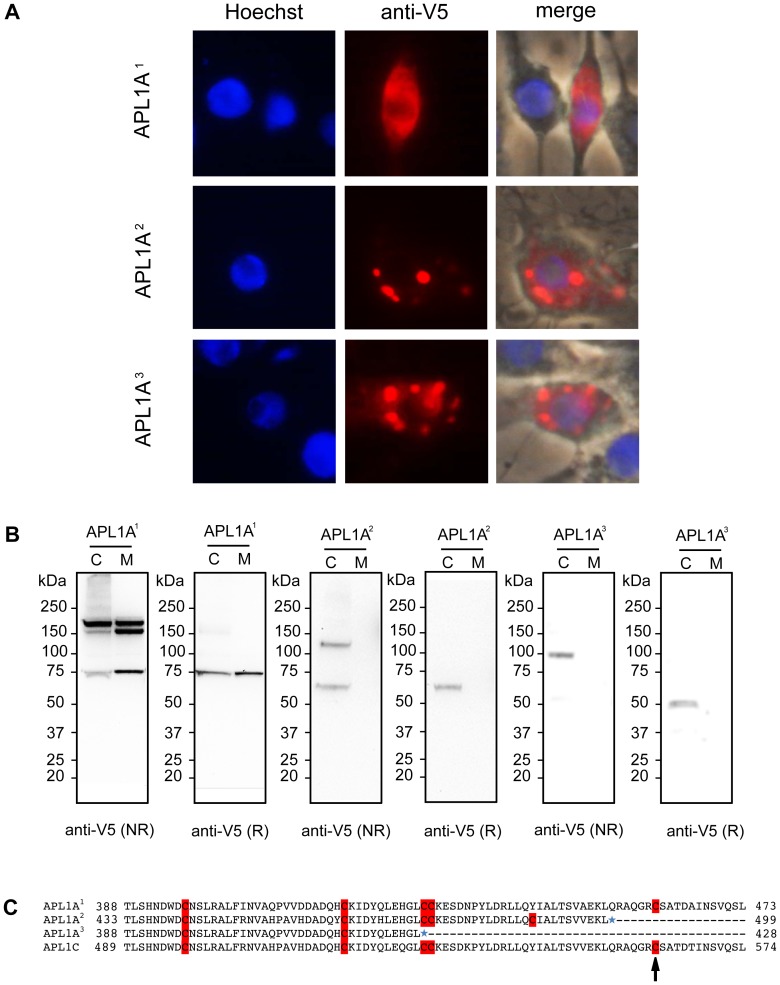
Differential secretion pattern and sub-cellular localization of APL1A-V5 alleles in hemocyte-like cell culture. A) Immunofluorescence analysis of 4a-3A hemocyte-like cells transfected with plasmids encoding V5-tagged APL1A^1^, APL1A^2^ and APL1A^3^. Cells were stained with Hoechst 33342 to label nuclei (blue). Staining with a mouse anti-V5 mAb followed by anti-mouse Alexa 488-conjugated IgG (red) indicates that APL1A^1^ exhibits a diffuse pattern in the cytoplasm whereas APL1A^2^ and APL1A^3^ are essentially localized in vesicles. Pictures were taken under equivalent exposure conditions. **B)** Immunoblot analysis of cells (C) and culture medium (M) of the 4a-3A hemocyte-like cell line transfected with plasmids encoding V5-tagged APL1A^1^, APL1A^2^ and APL1A^3^ under reducing (R) and non-reducing (NR) conditions. Immunoblots were probed with a mouse anti-V5 mAb, protein quantities on the different blots are not comparable. APL1A^1^ is secreted in the culture medium as at least two protein complexes under non-reducing conditions, whereas APL1A^2^ and APL1A^3^ are retained in the cell cytoplasm and form only one protein complex under non-reducing conditions. Estimated sizes of monomeric APL1A forms including V5-tag are: 76 kDa (APL1A^1^), 60 kDa (APL1A^2^) and 51 kDa (APL1A^3^). **C)** Amino acid alignment of cysteine-rich regions of the three Ngousso APL1A proteins and *A. gambiae* PEST APL1C. Numbers correspond to the amino acid positions. Blue stars represent stop codons, cysteine residues are highlighted in red. The cysteine residue corresponding to the position 562 in the APL1C sequence (APL1C^C562^, black arrow) described by Povelones *et al.* [18], is involved in the disulfide-linked complex formed between LRIM1 and APL1C and is only conserved in APL1A^1^. The same cysteine is also referred to as APL1C^ C551^ in the APL1C protein published by Baxter et al. [34].

The large structural differences between predicted APL1A protein isoforms (Figure 3) likely have functional consequences, which could explain the different protective profiles against *P. falciparum* infection. It is not possible to raise APL1A isoform-specific antibodies due to high peptide sequence similarity between the three structural variants. Thus, to examine protein function, we transfected the *A. gambiae* hemocyte-like cell line 4a-3A [25] with constructs expressing either APL1A^1^, APL1A^2^ or APL1A^3^ bearing a C-terminal V5-tag. Immunostaining of transfected cells with anti-V5 monoclonal antibody (mAb) showed that the weakly protective APL1A^1^ isoform exhibited a diffuse distribution throughout the cytoplasm, while the protective APL1A^2^ and non-protective APL1A^3^ isoforms were essentially localized within large vesicle-like structures (Figure 5A).

Comparison of protein processing by immunoblotting of washed cells and culture medium revealed that APL1A^1^ was constitutively secreted into the culture medium, while APL1A^2^ and APL1A^3^ were retained in the cell cytoplasm (Figure 5B). The differential constitutive secretion is even more striking given that all proteins carry a classical N-terminal peptide signal sequence [21]. Under non-reducing conditions, APL1A^1^ formed two high molecular weight complexes, consistent with predicted sizes of homodimer and heterodimer, in addition to the monomeric form expected at 76 kDa (Figure 5B, left panel). In contrast, APL1A^2^ and APL1A^3^ only formed complexes with sizes consistent with homodimers. Their inability to form heterodimers with other partners may be consistent with the absence of a C-terminal coiled-coil domain, and, more importantly, with the absence of a cysteine residue corresponding to the Cys-562 of APL1C (AGAP007033), which is responsible for the disulfide-bridged complex formed between LRIM1 and APL1C (Figure 5C) [18], [25]. These results suggest that the secretion of APL1A proteins may be dependent on domain interactions with binding partners. Interestingly, because these alleles are all found in nature, the observed polymorphism for the presence of a cysteine in APL1A equivalent to the APL1C^C562^ and consequent variation in protein partner interaction must be adaptive for APL1A function in the organism.

## Discussion

Natural populations of *A. gambiae* display such high genetic diversity and low linkage disequilibrium that it will be difficult to carry out genetic association studies using the wild population [26], [27]. This is particularly true for highly polymorphic genes, such as the *P. falciparum*-protective *APL1A*, coupled with the inherently noisy phenotype of *P. falciparum* infection [21]. Instead, it may be more efficient to perform initial variant discovery in a defined context that captures limited natural variation, followed by validation in the field.

In the current work, we show that the Ngousso colony has captured a repertoire of natural allelic variants of the *APL1* paralogs that result in structurally different proteins. The structurally most polymorphic paralog is *APL1A*, with three different alleles in Ngousso. The three *APL1A* alleles exist in nature, demonstrating that they are not laboratory colony mutations. Two alleles, *APL1A^1^* and *APL1A^2^*, are also found in the *A. gambiae* G3 colony [15], [21], which is much older than Ngousso and was sampled from a different geographic location in Africa [28]. Thus, the same *APL1A* alleles found in Ngousso have existed in nature over both space and time.

*A. gambiae* colonies are expected to segregate longer haplotypes than found in nature due to small effective population size and the founder effect, and we confirmed the haplotype structure of the *APL1* locus in the Ngousso colony. Consequently, in order to specifically measure the relative contribution of the three *APL1A* alleles to protection against *P. falciparum*, we globally silenced all variants of the *APL1A* gene, determined individual phenotypes for *P. falciparum* infection, and genotyped mosquitoes for *APL1A* using a semi-automated assay. When *APL1A* is silenced, the *APL1A^2^* allele accounts for essentially all of the ds*APL1A* effect on *P. falciparum* infection prevalence. The *APL1A^1^* allele has weak or no effect, and *APL1A^3^* has no influence on infection outcome. We did not detect an association between the haplotype bearing the *APL1* locus and infection outcome, but the haplotypes have captured linked variation for an unknown number of genes, including ones that may be able to influence infection phenotype.

The considerable structural differences of APL1 proteins, particularly of APL1A, suggest that the isoforms may interact with distinct protein partners. This hypothesis is supported by the different migration profiles of APL1A isoforms under non-reducing conditions. The APL1C protein forms a complex with LRIM1 via conserved cysteine residues, and this complex can bind, through the coiled-coil domain, to certain TEP proteins [17], [18]. Interestingly, APL1A^2^ and APL1A^3^, which lack a coiled-coil domain, form only one type of dimer under non-reducing conditions and are retained in the cytoplasm of hemocyte-like cells, whereas APL1A^1^, which has a coiled-coil domain and forms at least two different dimers, is secreted from the cell. The observed dimers of APL1A^2^ and APL1A^3^ show a size consistent with homodimers, though we cannot rule out that they could be heterodimers with unknown binding partners of similar molecular weight.

The mechanism underlying APL1A^1^ secretion remains elusive and it is not clear whether the secretion pattern is related to protection against *Plasmodium* infection. The behavior of APL1A alleles in culture does not necessarily translate to what happens *in vivo*. For example, absence of secretion of APL1A^2^ and APL1A^3^ may indicate that these alleles are not correctly folded in 4a-3A cells or that epitope tagging impairs their secretion. In addition, the APL1A^3^ allele lacks the Cys-Cys pair common to the entire LRIM family of LRR proteins (highlighted in Figure 5C) and this motif has been experimentally shown to be important for LRIM1 folding and secretion [18]. This suggests that APL1A^3^ may not be correctly folded and secreted even *in vivo*. Moreover, if APL1A secretion is dependent on interaction with binding partners, the putative APL1A^2^ and APL1A^3^ binding partners may not be expressed in 4a-3A cells. An alternative hypothesis could be that APL1A^1^, APL1A^2^ and APL1A^3^ are secreted through distinct mechanisms, and that the variants participate in resistance against *Plasmodium* in different ways. *In vivo*, APL1A^1^ might be constitutively secreted, while APL1A^2^ and APL1A^3^ secretion might be stimulated by immune elicitors or other factors. The potentially constitutive presence of APL1A^1^ in the hemolymph, if associated with a long protein half-life, could then explain why gene silencing experiments did not show a consistent protective effect for the *APL1A^1^* allele. Finally, another hypothesis is that APL1A^2^ functions as a dominant negative inhibitor of a factor required for *P. falciparum* development. Further work will be necessary to distinguish between these possibilities.

Our findings highlight the need to consider allelic variation of putative immune factors, including the spectrum of alleles segregating in a colony, in studies aimed at deciphering their function. We also demonstrate the utility of performing a discovery step in a recent colony that segregates a defined set of allelic variants that can be confirmed to exist in nature. The long haplotypes in colonies limit resolution, so in general it may not be meaningful to directly test genotype-phenotype association in colonies, because the allele genotypes are just a proxy for the longer haplotype. Rather, it is best to distinguish the effects of the candidate gene effects, and the influence of the carrying haplotype, either by allele-specific silencing when possible [22], or if the alleles offer insufficiently distinct dsRNA targets as for *APL1A*, by global gene silencing with allele-specific genotyping as we did here. The information gained should then permit the design of statistically well-powered tests of small numbers of candidate variants in the natural population, where resolving power is greater due to the low linkage disequilibrium. Based on the current findings, it would be interesting to directly compare phenotypic outcomes of *APL1A^1^, APL1A^2^* and *APL1A^3^* carriers in the wild population after challenge with *P. falciparum*.

## Materials and Methods

### Mosquito Rearing

The *A. gambiae s.s.* colony Ngousso was established with mosquitoes captured in Yaounde, Cameroon in January 2006 [29]. The mosquitoes are of the M molecular and Forest chromosomal form, fixed for the standard 2La chromosomal inversion. The colony was reared at the CEPIA mosquito production facility at the Institut Pasteur under standard rearing conditions at 26°C and 80% relative humidity, under a 12 h light/dark cycle as described elsewhere [29].

### *P. falciparum* Gametocyte Culture and Ngousso Infection

Parasite culture and experimental feedings with the *P. falciparum* isolate NF54 were done as previously described [11]. Briefly, *P. falciparum* NF54 was cultured using the automated tipper-table system of Ponnudurai [30] implemented in the CEPIA mosquito facility of Institut Pasteur. Fourteen days after initiating the parasite subculture and prior to each infection experiment, gametocyte maturity was assessed by testing exflagellation of male microgametes. Gametocytaemia and proportions of mature male and female gametocytes were determined on Giemsa stained slides (Table S2).

For an infectious blood meal, 10 ml of the gametocyte culture were then centrifuged at 2000 rpm, and the cell pellet was resuspended in an equal volume of normal type AB human serum. The infected erythrocytes were added to fresh erythrocytes in AB human serum and transferred into a membrane feeder warmed to 37°C. Female mosquitoes (4–5 days old) were allowed to feed for 15 min, unfed mosquitoes were removed and only fully engorged females were maintained on 10% sucrose solution for further analysis.

Each infection experiment replicate was done with a new generation of Ngousso females and a new gametocyte culture of *P. falciparum* NF54.

### Analysis of Infection Phenotypes

As infection phenotypes we analyzed prevalence and intensity. Infection prevalence is the fraction of mosquitoes carrying at least one oocyst, while parasite intensity is the number of oocysts per mosquito determined only in the subset of mosquitoes with ≥1 oocyst.

To determine infection phenotype, midguts of bloodfed females were dissected 7–8 days post-infection, stained in 1×PBS buffer with 0.4% mercury dibromofluorescein (Sigma) and the number of oocysts per midgut was determined using a light microscope. Carcasses of the dissected mosquitoes were immediately transferred individually into a fresh tube and stored at −20°C until DNA extraction. Amongst the infection experiments for the association study we analyzed only those trials where infection prevalence was between 35 and 55% (Table S2B).

In infection experiments with dsRNA injection, we retained only those trials where infection prevalence of mosquitoes treated with the control dsRNA, ds*GFP,* satisfied this criterion (Table S2A). Differences in infection prevalence were analyzed using the Chi-Square test. For intensity-related infection, non-parametric statistical tests were used, including the Mann-Whitney Rank Sum Test and the Kruskal-Wallis ANOVA on ranks, excluding mosquitoes with zero oocysts. Three independent replicate infections were performed and data were pooled prior to statistical analysis.

### DNA Preparation

Genomic DNA was extracted from individual female mosquitoes by homogenizing in 100 µl DNAzol (Invitrogen, CA, USA) using a disposable pestle, essentially following the manufacturer’s protocol.

### PCR Reactions

The *APL1* paralogs correspond to the VectorBase identifiers *APL1A* (*AGAP007036)*, *APL1B*, (*AGAP007035)* and *APL1C*, (*AGAP007033)*. PCR primers were designed based on the sequence of the *A. gambiae* PEST reference genome or on available Ngousso sequences. Primers were designed to bind in the exon regions of the *APL1* genes. Genomic DNA of a single mosquito was used for PCR and sequencing reactions. For evaluation of size differences of PCR fragments, amplification reactions were performed in a final volume of 20 µl using Taq DNA polymerase (Invitrogen). PCR cycles were as follows: a denaturation step at 94°C for 3 minutes, followed by 40 cycles of 94°C for 30 sec, 62°C for 45 sec, 72°C for 3 minutes and a final extension of 72°C for 10 minutes. Primers and their positions are given in Table S1.

DNA amplification for sequencing was done using AccuPrime SuperMixII (Invitrogen). Primers carried a 5′-extension for sequencing with the universal forward (–21 M13, 5′ TGT AAA ACG ACG GCC AGT 3′) or reverse (M13reverse, 5′ CAG GAA ACA GCT ATG ACC 3′) primers [31]. Final reaction volume was 50 µl and cycling conditions for amplification used denaturation at 94°C for 3 minutes, followed by 40 cycles at 94°C for 30 sec, 62°C for 45 sec and 72°C for 3 minutes and a final extension step of 72°C for 10 minutes.

### Sequencing

PCR products were sequenced using ABI Big Dye Terminator v.3.1 Cycle Sequencing kit (LifeTechnologie) and an ABI Prism 3730 DNA Analyzer (Applied Biosystems). The sequences were assembled using CodonCode Aligner (CodonCode Corporation). Heterozygous peaks were identified manually. Sequences were analyzed using eBioX/eBiotools [32]. Coordinates of features including lengths of the indels are based on the PEST reference sequence. *APL1* sequences were deposited into Genbank under accession numbers JX292981 to JX292986.

### Development of a RFLP Assay to Determine the *APL1A* Alleles in Ngousso

We developed a high-throughput Restriction Fragment Length Polymorphism test (RFLP) to facilitate the identification of the *APL1A* alleles present in Ngousso mosquitoes. This assay takes advantage of the allele specific indel patterns in the *APL1A* genes. These indel patterns are stable in the Ngousso colony, distinguish the *APL1A* allelic variants and provide information on the haplotype of the *APL1* region.

The *APL1A*-RFLP test is based on the PCR fragment spanning the complete *APL1A* coding-region. The full-length amplicon is generated with two fluorescent primers labeled with different fluorophores and then cleaved with *Pst*I. Resulting fluorescent-labeled restriction fragments, which span the indel bearing regions at the 5′ and 3′ end of the *APL1A* alleles (Figure 2) are distinguishable by size and color. Fragment separation and sizing were done on an ABI Prism 3730 DNA Analyzer. In combination, the two sized, end-labeled fragments provide information about the presence or absence of the PANGGL repeat region and determined the patterns of the small indels in the 3′region of the *APL1A* gene based on alterations of the restriction fragment length. The *APL1A*-RFLP distinguishes the three allelic *APL1A* variants present in the Ngousso laboratory colony and all combinatorial genotypes of the *APL1A* alleles were found in the colony.

### Double-stranded RNA Synthesis and Injection

Double-stranded RNAs were synthesized from PCR amplicons using the T7 Megascript Kit (Ambion) as described previously [11]. 500 ng of ds*APL1A* and ds*GFP* in a maximum volume of 207 nl were injected into the thorax of cold-anesthetized 1 day-old *A. gambiae* females using a nano-injector (Nanoject II; Drummond). Mosquitoes were challenged with *P. falciparum* parasites 4 days after dsRNA injection.

### Gene Knockdown Verification

The efficiency of transcript knockdown was monitored 4 days after dsRNA injection. cDNA synthesis was performed by using M-MLV reverse transcriptase and random hexamers (Invitrogen). In each case, 500 ng of total RNA was used in triplicate assays. The triplicates were pooled and the mixture was used as template for PCR analysis. Gene knockdown verification was performed as described in Mitri *et al.* 2009 [11].

### Insect Cell Culture and Transfection

*A. gambiae* derived 4a-3A hemocyte-like cells were cultured in monolayer at 27°C in Insect Xpress medium (Lonza) supplemented with 5% foetal bovine serum (GIBCO BRL) and 50 µg/ml gentamycin (Sigma). The three *APL1A* alleles were amplified from selected mosquitoes with known *APL1* haplotypes by PCR using the following primers flanking the coding regions of each gene: APL1A^1^5′EcoRI (5′ GGG AAT TCC CTG TTT CGA GTG CTA TAA TG 3′), APL1A^1^3′V5XbaI (5′ GGT CTA GAG TTA GGT CTG TGA TTG GCG AG 3′), APL1A^2^5′EcoRI (5′ GGG AAT TCC GAG CTT TGA GTA CCA CAA TG 3′), APL1A^2^3′V5XbaI (5′ CCT CTA GAC AAC TTC TCC ACC ACG CTC 3′), APL1A^3^5′EcoRI (5′ GGG AAT TCC CTG TTT CGA GTG CTA TAA TG 3′), APL1A^3^3′V5XbaI (5′ GGT CTA GAG AGA CCA TGC TCG AGT TGG 3′).

The amplicons were cloned into a dual His and V5-tag insect expression vector (pAc5.1 V5/His, Invitrogen) or a dual Strep and V5-tag insect expression vector (pAc5.1 V5/Strep). The pAc5.1 V5/Strep vector is a variant of the pAc5.1 V5/His insect expression vector that was modified to replace the His-tag by a Strep-tag. 4a-3A cells were co-transfected with the expression vector and the selection vector pCoBlast Puromycin in a ratio 9∶1 with Cellfectin II reagent (Invitrogen) according to the manufacturer’s protocol. Three days after transfection, antibiotic selection was started with 6 µg/ml puromycin (Invitrogen). After one month the cell population was tested for expression.

### Western Blotting

To analyze the secretion pattern for tagged proteins, the culture medium was collected and spun for 10 min at 800 rpm to remove floating cells and large debris. Adherent cells were washed in 1×PBS and collected by scraping in 10 mM Tris pH 8. Proteins from culture medium and cells were extracted in XT sample buffer (Bio-Rad), heated at 95°C for 5 min and separated on 4–12% Criterion SDS-PAGE gels (Bio-Rad). Reduced samples were prepared by adding XT reducing agent (Bio-Rad) before heating. After protein transfer to PVDF membrane, immunoblots were blocked for 1 hour in 5% nonfat dry milk, probed with a mouse mAb anti-V5 antibody at 1∶5000, followed by 1 hour with horseradish peroxidase-conjugated anti-mouse IgG secondary antibodies (Promega) at 1∶10 000. The detection step was performed using the Pierce chemiluminescence system (Pierce) following the manufacturer’s instructions.

### Immunofluorescence Assays

Transfected cells were allowed to grow in an 8-well Lab-Tek chamber slide system (Thermo Scientific) for one hour. The culture medium was then removed, the cells were washed in 1×PBS, fixed in 1×PBS with 4% paraformaldehyde for 30 min at room temperature, and permeabilized in 0.2% Triton X-100 and 5% Bovine Serum Albumin in PBS 1X for 45 min at room temperature. Cells were incubated with a mouse mAb anti-V5 antibody at 1∶500 in 1×PBS containing 10% foetal bovine serum for 1 hour at 4°C, followed by 1 hour with Alexa Fluor 594-conjugated anti-mouse IgG secondary antibody at 1∶2000 and Alexa Fluor 488-conjugated phalloidin (Molecular Probes) at 1∶500 in PBS 1X. Nuclei were stained with Hoechst 33342. After mounting in SlowFade Gold antifade reagent (Molecular Probes), samples were observed at 100×magnification using a Leica DM 5000 B fluorescent microscope.

## Supporting Information

Figure S1 **Alignment of genomic regions coding for** ***APL1A, APL1B*** **and** ***APL1C*** **alleles in Ngousso mosquitoes.** Nucleotide sequences have been aligned using ClustalW. Prettyplot (EMBOSS package) has been used for boxing and colouring with plurality = 4 to calculate the consensus. Red colour indicates nucleotides identical to the consensus. Start codons of the *APL1* alleles are highlighted in blue and the stop codons in green. The deletions-insertions described in Supplementary Information S1 (Structure of *APL1* alleles in Ngousso) are highlighted in yellow. Stars indicate borders of the nucleotide sequences used in the polydot blots of Figure S5. The 5′ end position is common to all alleles and corresponds to the first nucleotide of the start codon. At the 3′ end a black star indicates the end of the fragment for *APL1A* and *APL1B* alleles and red star the end of the *APL1C* alleles. Localizations of the oligonucleotides used to generate the dsRNA are indicated by arrows.
(PDF)

Figure S2 **Observed infection intensities in silencing experiments.** The experiment number (Exp.1, Exp.2 and Exp.3) and the RNAi knockdown target are shown on the horizontal axis. The number of infected mosquitoes (n) from each knockdown experiment is indicated. *GFP*kd was used as a control dsRNA. The vertical axis shows the number of midgut oocysts 7–8 days following a *P. falciparum* infectious blood meal. The median number of oocysts is indicated by the solid horizontal bar. The calculated *p*-values, comparing *GFP*kd with *APL1A*kd, indicate lack of a statistically significant effect of *APL1A* silencing on infection intensity in the three independent experiments.
(PDF)

Figure S3 **Observed infection intensities without knockdown.** The experiment number (Inf1, Inf2 and Inf3) is shown on the horizontal axis. The number of infected mosquitoes (n) from each experiment is indicated. The vertical axis shows the number of midgut oocysts 7–8 days following a *P. falciparum* infectious blood meal. The median number of oocysts is indicated by the solid horizontal bar.
(PDF)

Figure S4 **Determination of** ***APL1A*** **allele frequencies and genotypes in the Ngousso population with the** ***APL1A*** **-RFLP test. A) Observed** ***APL1A*** **allele frequencies.** The histogram shows the allele composition (in percentage) of Ngousso females in three independent infection experiments (Inf1, Inf2 and Inf3). Numbers below the x-axis correspond to the alleles *APL1A^1^* (1), *APL1A^2^* (2) and *APL1A^3^* (3), respectively. **B) Observed** ***APL1A*** **genotype frequencies.** The histogram shows the *APL1A* genotype composition (in percentage) of all Ngousso females from the three infection experiments (Inf1, Inf2 and Inf3) analyzed in figure S4A. Numbers below the x-axis correspond to following genotypes: *APL1A^1^/APL1A^1^* (1-1), *APL1A^1^/APL1A^2^* (1-2), *APL1A^1^/APL1A^3^* (1-3), *APL1A^2^/APL1A^2^* (2-2), *APL1A^3^/APL1A^2^* (3-2) and *APL1A^3^/APL1A^3^* (3-3).
(PDF)

Figure S5 **Polydot plot of** ***APL1*** **genes and their allelic variants.** Polydot software [2] has been used to perform pair wise comparisons of all Ngousso (Ng) *APL1* alleles or their encoded proteins in order to illustrate their high degree of similarity. The word size used for the plots is indicated on top of the graphs. It corresponds to the length of the fragment that should have an exact match in both sequences used in the comparison. **A: Polydot plot of** ***APL1*** **alleles.** For the comparison of all *APL1* alleles in Ngousso, the corresponding genomic regions have been extracted, beginning at their start codon and up to the position of the stop codon of the longest allele of each gene, i.e.: *APL1A* (2L:41270938.41270940), *APL1B* (2L:41266619.41266621) and *APL1C* (2L:41257877.41257879). Red boxes highlight comparisons between alleles of the same gene (*APL1A*, *APL1B* of *APL1C*) and therefore show the alignments with the highest identity level. For *APL1B* and *APL1C* the nucleotide identity is high over the complete gene while for *APL1A* the differences between the alleles are visible: *APL1A^1^* and *APL1A^3^* display a nearly perfect diagonal while the *APL1A^2^* allele harbors a different 5′end due to the repeat region which is similar to the *APL1C* alleles. It should be noted that the most divergent part on this plots between *APL1A* genes and *APL1B*/*APL1C* genes is the 3′ end where the dsRNA has been choosen (see Figure S1C for more details). **B: Polydot plot of APL1 protein variants.** The protein sequences of all APL1 variants were used in the comparison. To permit the comparison with the very short *APL1B^3^* allele, its premature stop codon has been ignored, resulting in an artificial *in silico* product, which was extended to the same length as the other APL1B proteins. **C: Polydot plot of** ***APL1*** **genomic regions against the nucleotide sequence of the** ***APL1A*** **dsRNA regions.** Oligonucleotides used for the synthesis of the ds*APL1A* are positioned at (2L: 41271198.41271218) and downstream of the *APL1A^1^* stop codon (2L:41270907.41270930). For this comparison the corresponding sequence of the 3′end of each *APL1A* allele was extracted and compared to the sequence of the PCR fragments of the entire *APL1* genes. To illustrate the specificity of the dsRNA used in the knock-down experiments, two different word sizes were used. With a word size of 20, corresponding to the size range of RNAi fragments, exact matches can only be found with the *APL1A* genes. When applying less stringent conditions by reducing the word size to 8, still no obvious homology with the *APL1B* and *APL1C* genes can be detected, demonstrating the specificity of the ds*APL1A* used in the knock-down experiments.
(PDF)

Table S1 **Oligonuclotides used to amplify** ***APL1*** **genes and genomic regions from** ***A. gambiae*** **Ngousso.**
(PDF)

Table S2 **Parameters of the** ***P. falciparum*** **gametocyte (NF54) cultures and results of the different infection experiments.**
(PDF)

Supplementary Information S1 (PDF)
