# Supplementary material for: Diverged Alleles of the Anopheles gambiae Leucine-Rich Repeat Gene APL1A Display Distinct Protective Profiles against Plasmodium falciparum
Source: PLoS One. 2012 Dec 28;7(12):e52684. doi: 10.1371/journal.pone.0052684 (PMC3532451; doi:10.1371/journal.pone.0052684)
Supplement: Figure S1 — Alignment of genomic regions coding for APL1A, APL1B and APL1C alleles in Ngousso mosquitoes. Nucleotide sequences have been aligned using ClustalW. Prettyplot (EMBOSS package) has been used for boxing and colouring with plurality = 4 to calculate the consensus. Red colour indicates nucleotides identical to the consensus. Start codons of the APL1 alleles are highlighted in blue and the stop codons in green. The deletions-insertions described in Supplementary Information S1 (Structure of APL1 alleles in Ngousso) are highlighted in yellow. Stars indicate borders of the nucleotide sequences used in the polydot blots of Figure S5. The 5′ end position is common to all alleles and corresponds to the first nucleotide of the start codon. At the 3′ end a black star indicates the end of the fragment for APL1A and APL1B alleles and red star the end of the APL1C alleles. Localizations of the oligonucleotides used to generate the dsRNA are indicated by arrows. (PDF) [file pone.0052684.s001.pdf]

Figure S1: Alignment of genomic regions coding for *APL1A*, *APL1B* and *APL1C* alleles in Ngousso mosquitoes

```
Ng_APL1A_1   TTTAAACACCAACATATCCTGTGTCACGTTGGAGAGCGAATTTACTCAATCATAACATTTGTTTACATATTCAAG--ACGCCGTTATCTCTGGGAATTCCCGT----------TCTTTCA   108
Ng_APL1A_2   GTGCTGTTGGAACATATCCTGTACCGCGTTGGAGAGCAAATCAACTCAATCATAACATTTGTTTACATATTCAAG--ACGCTGTTATCTCTGGGAATTTCCGT----------TCTTTCA   108
Ng_APL1A_3   TTTAAACACCAACATATCCTGTGTCACGTTGGAGAGCGAATTTACTCAATCATAACATTTGTTTACATATTCAAG--ACGCCGTTATCTCTGGGAATTCCCGT----------TCTTTCA   108
Ng_APL1B_1   ---AAACACCGATAAGTACTATGACA---------GTGAATTTACTCAATCATAACATTTGTTTTCATTTTCACGCCACGCCATTATCACTGGGTATTTCCGTAACGAATTCTTCTCTCA   108
Ng_APL1B_2   ---AAACACCGATAAGTACTATGACA---------GTGAATTTACTCAATCATAACATTTGTTTTCATTTTCACGCCACGCCATTATCACTGGGTATTTCCGTAACGAATTCTTCTCTCA   108
Ng_APL1B_3   ---AAACACCGATAAGTACTATGACA---------GTGAATTTACTCAATCATAACATTTGTTTTCATTTTCACGCCACGCCATTATCACTGGGTATTTCCGTAACGAATTCTTCTCTCA   108
Ng_APL1C_1   --------CCGGCAAGTGCTGGGCCGCGTTGGTG--CGAATTTACTCAATCATAACATTTGTTTTCATTTTCAGGA--CGCCGTTATCACTGGGAATTCCCGTAACGAATTCATCTTTCA   108
Ng_APL1C_2   --------CCGGCAAGTGCTGGGCCGCGTTGGTG--CGAATTTACTCAATCATTACATTTGTTTTCATTTTCAGGA--CGCCGTTATCACTGGGAATTCCCGTAACGAATTCATCTTTCA   108
Ng_APL1C_3   --------CCGGCAATTGCTGGGCAGCGTTGGTG--CGAATTTACTCAATCATTACATTTGTTTTCATTTTCAGGA--CGCCGTTATCACTGGGAATTCCCGTAACGAATTCATCTTTCA   108

                                                                                                 ★
Ng_APL1A_1   GTCAGCTGAGCATGTTCCACCGTCGAAATCATTGCTCAGTTTCAATCCATCAGTCGCGGAAGAAACACAGTTTTCCTGTTTCGAGTGCTATAATGTGCTGGTTACGCGCCGTATCACTAA   228
Ng_APL1A_2   GTCAGCTGAGCATGTTCCACCGTCGGAATCATTGCTCAGTTTCAATCCATCAGTCGCGGAAGAAACACAGTTGTCGAGCTTTGAGTACCACAATGTGCGGGTTACGCGCCGTATCACTAA   228
Ng_APL1A_3   GTCAGCTGAGCATGTTCCACCGTCGAAATCATTGCTCAGTTTCAATCCATCAGTCGCGGAAGAAACACAGTTTTCCTGTTTCGAGTGCTATAATGTGCTGGTTACGCGCCGTATCACTAA   228
Ng_APL1B_1   GTCGGCTGAGCAAGTTCTACCGTAGGTGTCATTGCACAGTTTCAATCCATCAGTCGAAGAAGAAACACAGTTTTTCTGTTTTGAGTGCCACAATGTGCTGGTTACGCGCCGTATCTCTAA   228
Ng_APL1B_2   GTCGGCTGAGCAAGTTCTACCGTAGGTGTCATTGCACAGTTTCAATCCATCAGTCGAAGAAGAAACACAGTTTTTCTGTTTTGAGTGCCACAATGTGCTGGTTACGTGCCGTATCACTAA   228
Ng_APL1B_3   GTCGGCTGAGCAAGTTCTACCGTAGGTGTCATTGCACAGTTTCAATCCATCAGTCGAAGAAGAAACACAGTTTTTCTGTTTTGAGTGCCACAATGTGCTGGTTACGTGCCGTATCACTAA   228
Ng_APL1C_1   GTGTGCTACGCTAGTTCTACCGCAGGAATGATTGCTCAGTTTCAATCCATCATTCGAGGAAGAAACACAGTTTTCGAGCATTGAGTACCACAATGTGCTGGGTACGCGCCGTATCACTAA   228
Ng_APL1C_2   GTGTGCTACGCTAGTTCTACCGCAGGAATGATTGCTCAGTTTCAATCCATCGTTCGAGGAAGAAACACAGTTTTCGAGCATTGAGTACCACAATGTGCTGGTTACACGCCGTATCACTAA   228
Ng_APL1C_3   GTGTGCTACGCTAGTTCTACCGCAGGAATGATTGCTCAGTTTCAATCCATCATTCGAGGAAGAAACACAGTTTTCGAGCATTGAGTACCACAATGTGCTGGTTACACGCCGTATCACTAA   228

Ng_APL1A_1   TAAGGTAAAGCTCAGCAATCTATAGTTA----TTACTACACATCGCTT-ACAGAGTGTGTATTTTTGTGTGGGTCACTGCTTTTGCAGTGTAATTTTTTT--------------------   323
Ng_APL1A_2   TCAGGTAATTGTCAGCAGTGTACAGTTACGTATTACTTCATATCGCTCAACAGAGTATTTCTTTTT-TGTCGGTTGCTGCTTTGGCAGTGTGATTTTCTTCACATGTGGTAGTGTTGCAG   347
Ng_APL1A_3   TAAGGTAAAGTTCAGCAATCTATAGTTA----TTACTACACATCGCTT-ACAGAGTGTGTATTTTTGTGTGGGTCACTGCTTTTGCAGTGTAATTTTTTT--------------------   323
Ng_APL1B_1   TCAGGTAACGCTCAGCATCAAACAATTAGGCATTACTTCATATCGCTT-ACAGAGTGTTTCTTTGT-TGTTGGTTACTG-TATTGCAGTATGATCTTTTC--------------------   325
Ng_APL1B_2   TCATTTAACGCTCAGCATCAAACAATTAGGCATTACTTCATATCGCTT-ACAGAGTGTTTCTTTGT-TGTTGGTTACTG-TATTGCAGTATGATCTTTTC--------------------   325
Ng_APL1B_3   TCAGGTAACGCTCAGCATCAAACAATTAGGCATTACTTCATATCGCTT-ACAGAGTGTTTCTTTGT-TGTTGGTTACTG-TATTGCAGTATGATCTTTTC--------------------   325
Ng_APL1C_1   TCAGGTAATTGTCAGCAGTGTACATTTACGTATTACTTCATATCACTTAACAGTGTGTTTCTTTTT-TGTCGATTGCTGCTTTGGCAGTGTGATTTTCTTCACATGTGGTAGTGTTGCAG   347
Ng_APL1C_2   TCAGGTAATTGTCAGCAGTGTACAGTTACGTATTACTTCATATCACTTAACAGTGTGTTTCTTTTT-TGTCGATTGCTGCTTTGGCAGTGTGATTTTCTTCACATGTGGTAGTGTTGCAG   347
Ng_APL1C_3   TCAGGTAATTGTCAGCAGTGTACAGTTACGTATTACTTCATATCACTTAACAGTGTGTTTCTTTTT-TGTCGATTGCTGCTTTGGCAGTGTGATTTTCTTCACATGTGGTAGTGTTGCAG   347

Ng_APL1A_1   ------------------------------------AGCCTGT-----------------------------------------------------------------------------   330
Ng_APL1A_2   CTGTTGGGGGCAATAATTATTGGTATCCGAAAGAGCAGCCTGTTGCGACCACCAAACCTTCTTTCCCTGGGAACGGATACTACATCTACGGAAGTCAGCCAGCCAACGGTGGTCTGCCAG   467
Ng_APL1A_3   ------------------------------------AGCCTGT-----------------------------------------------------------------------------   330
Ng_APL1B_1   ------------------------------------AGCCAGT-----------------------------------------------------------------------------   332
Ng_APL1B_2   ------------------------------------AGCCAGT-----------------------------------------------------------------------------   332
Ng_APL1B_3   ------------------------------------AGCCAGT-----------------------------------------------------------------------------   332
Ng_APL1C_1   CTGTTGGGGGCAATAATTATTGGTATACGGAAGAGCAGCCTGTTGCGACCACCAAACCTTCTTTCCATGTGAACGGATACAACATCTACGGAAGTCAGCCAGCCAACGGTGGTCTGCCAG   467
Ng_APL1C_2   CTGTTGGGGGCAATAATTATTGGTATACGGAAGAGCAGCCTGTTGCGACCACCAAACCTTCTTTCCATGTGAACGGATACAACATCTACGGAAGTCAGCCAGCCAACGGTGGTCTGCCAG   467
Ng_APL1C_3   CTGTTGGGGGCAATAATTATTGGTATACGGAAGAGCAGCCTGTTGCGACCACCAAACCTTCTTTCCATGTGAACGGATACAACATCTACGGAAGTCAGCCAGCCAACGGAGGTC------   461
```

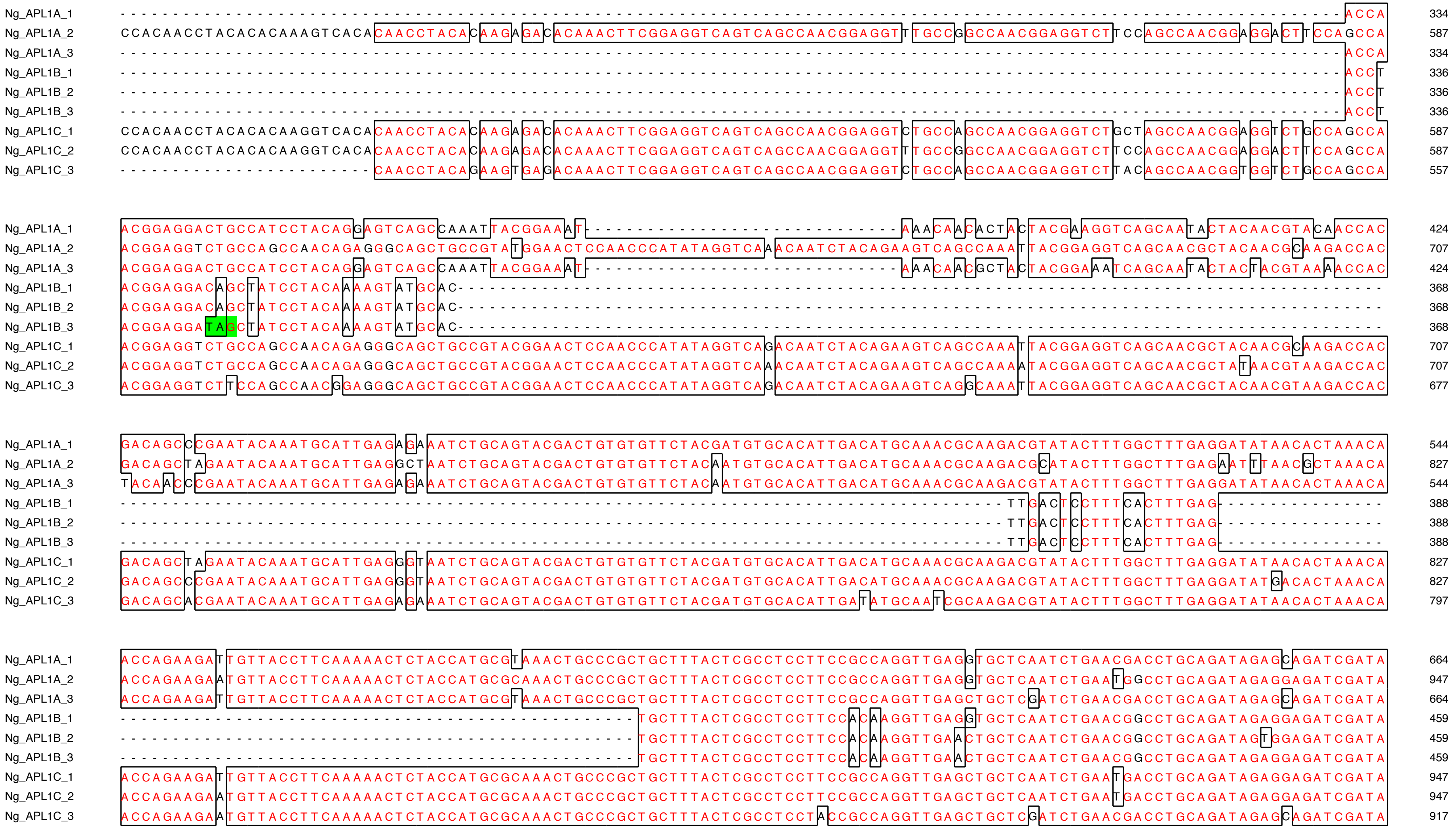
Ng_APL1A_1
Ng_APL1A_2
Ng_APL1A_3
Ng_APL1B_1
Ng_APL1B_2
Ng_APL1B_3
Ng_APL1C_1
Ng_APL1C_2
Ng_APL1C_3
334
587
334
336
336
336
587
587
557
424
707
424
368
368
368
707
707
677
544
827
544
388
388
388
827
827
797
664
947
664
459
459
459
947
947
917

```
Ng_APL1A_1   CGAACGCGTTCGCTTACGCGCACACAATTCAGAAGCTGTACATGGGATTCAATGCGATTCGATATTTGCCACCGTACGTGTTTCAGAACGTCCCATCGCTCACGGTGCTTGTGCTGGAAC   784
Ng_APL1A_2   CGAACGCGTTCGCTTACGCGCACACAATACAGAAGCTGT---------------------------------------------------------------------------------   986
Ng_APL1A_3   CGAACGCGTTCGCTTACGCGCACACAATTCAGAAGCTGTACATGGGATTCAATGCGATTCGATATTTGCCACCGTACGTGTTTCAGAACGTCCCATCGCTCACGGTGCTTGTGTTGGAAC   784
Ng_APL1B_1   CGAACGCGTTTGCTTACGCGCACACAATACAGAAGCTGTACATGAGATTCAATGTGATTAGATATTTGCCACCACACGTGTTTCAGAACGTCCCACTGCTCACGGTGCTTATGCTGGACC   579
Ng_APL1B_2   CGAACGCGTTCGCTTACGCGCACACAATACAGAAGCTGTACATGAGATTCAATGTGATTCGATATTTGCCACCGCACGTGTTTCAGAACGTCCCACTGCTCACGGTGCTTATGCTGGACC   579
Ng_APL1B_3   CGAACGCGTTCGCTTACGCGCACACAATACAGAAGCTGTACATGAGATTCAATGCGATTCGATATTTGCCACCGCACGTGTTTCAGAACGTCCCATCGCTCACGGTGCTTGTGTTGGAAC   579
Ng_APL1C_1   CGAACGCGTTCGCTTACGCGCACACAATTCAGAAGCTGTACATGGGATTCAATGCGATTCGATATTTGCCACCGTACGTGTTTCAGAACGTCCCACTGCTCACGGTGCTTGTGCTGGAAC   1067
Ng_APL1C_2   CGAACGCGTTCGCTTACGCGCACACAATTCAGAAGCTGTACATGGGATTCAATGCGATTCGATATTTGCCACCGTACGTGTTTCAGAACGTCCCACTGCTCACGGTGCTTGTGCTGGAAC   1067
Ng_APL1C_3   CGAACGCGTTCGCTTACGCGCACACAATACAGAAGCTGTACATGGGATTCAATGCGATTCGATATTTGCCACCGTACGTGTTTCAGAACGTCCCATCGCTCACGGTGCTTGTGCTGGAAC   1037

Ng_APL1A_1   GGAACGATTTAACCAGCCTGCCACGCGGAATCTTCCACAACACACCCAAGCTCAGCATGCTGTCGATGTCAAACAACAACCTGGAGCGCATCGAAGACGAAACTTTCCAAGCAACTACAA   904
Ng_APL1A_2   ---------------------------------------------------------------CAATGTCGAACAACAACCTGGAGCGCATCGAAGACGACACTTTCCAAGCGACTACAG   1043
Ng_APL1A_3   GGAACGATTTAACCAGCCTGCCACGCGGAATCTTCCACAACACACCCAAGCTTACCATGCTGTCGATGTCAAACAACAACCTGGAGCGCATCGAAGACGAAACTTTCCAAGCAACTACAA   904
Ng_APL1B_1   GGAACGATTTATCCAGCCTGCCACCCGGAATCTTCCACAACACACCCAAGCTCACCATGCTGTCGATGTCAAACAACAACCTGGAACGCATCGAAGACGACACTTTCCAAGCGACTACAT   699
Ng_APL1B_2   GGAACGATTTATCCAGCCTGCCACGCGGAATCTTCCACAACACACCCAAGCTCACCATGCTGTCGATGTCAAACAACAACCTGGAGCGCATCGAAGACGAAACTTTCCAAGCGACTACAG   699
Ng_APL1B_3   GGAACGATTTAACCAGCCTGCCACGCGGAATCTTCCACAACACACCCAAGCTCACCATGCTGTCGATGTCAAACAACAACCTGGAGCGCATCGAAGACGACACTTTCCAAGCGACTACAG   699
Ng_APL1C_1   GGAACGATTTAACCAGCCTGCCACGCGGAATCTTCCACAACACACCCAAGCTCAGCATGCTGTCGATGTCAAACAACAACCTGGAGCGCATCGAAGACGACACTTTCCAAGCGACTACAG   1187
Ng_APL1C_2   GGAACGATTTAATCAGCCTGCCACGCGGAATCTTCCACAACACACCCAAGCTCAGCATGCTGGCGATGTCGAACAACAACCTGGAACGCATCGAAGACGACACTTTCCAAGCGACTACAG   1187
Ng_APL1C_3   GGAACGATTTAACCAGCCTGCCACGCGGAATCTTCCACAACACACCCAAGCTCACCATGCTGTCGATGTCAAACAACAACCTGGAGCGCATCGAAGACGACACTTTCCAAGCGACTACAG   1157

Ng_APL1A_1   CGCTTCAAAATCTTCAACTGTCCAGCAACAGGCTTACGCATGTTGACCTTTCTCTCATTCCCAGTCTCTTCCACGTCAACGTCAGTTACAACCTGCTCTCAACGCTTGCCATACCGATCG   1024
Ng_APL1A_2   CGCTTCAAAATCTGCAACTGTCCAGCAACAGGCTTACGCATGTTGACCTTTCTCTCATTCCCAGTCTCTTCCACGTCAACGTCAGTTACAACCTGCTCTCTACACTTGCCATACCGATCG   1163
Ng_APL1A_3   CGCTTCAAAATCTTCAACTGTCCAGCAACAGGCTTACGCATGTTGACCTTGCTCTCATTCCCAGTCTCTTCCACGTCAACGTCAGTTACAACCTGCTCTCAACGCTTGCCATACCGATCG   1024
Ng_APL1B_1   CGCTTCAAAATCTTCAACTGTCCAGCAACAGGCTTACGCATGTTGACCTTGCTCTCATTCCCAGTCTCTTCCACGTCAACGTCAGTTACAACCTGCTCTCAACGCTTGCCATACCGATCG   819
Ng_APL1B_2   CGCTTCAAAATCTTCAACTGTCCAGCAACAGGCTTACGCATGTTGACCTTGCTCTCATTCCCAGTCTCTTCCACGTCAACGTCAGTTACAACCTGCTCTCAACGCTTGCCATACCGATCG   819
Ng_APL1B_3   CGCTTCAAAATCTTCAACTGTCCAGCAACAGGCTTACGCATGTTGACCTTGCTCTCATTCCCAGTCTCTTCCACGTCAACGTCAGTTACAACCTGCTCTCAACGCTTGCCATACCGATCG   819
Ng_APL1C_1   CGCTTCAAAATCTGCAACTGTCCAGCAACAGGCTTACGCATGTTGACCTTTCTCTCATTCCCAGTCTCTTCCACGTCAACGTCAGTTACAACCTGCTCTCTACGCTTGCCATACCGATCG   1307
Ng_APL1C_2   AGCTTCAAAATCTTCAACTGTCCAGCAACAGGCTTACGCATGTTGACCTTGCTCTCATTCCCAGTCTCTTCCACGTCAACGTCAGTTACAACCTGCTCTCAACGCTTGCCATACCGATCG   1307
Ng_APL1C_3   CGCTTCAAAATCTGCAACTGTCCAGCAACAGGCTTACGCATGTTGACCTTTCTCTCATTCCCAGTCTCTTCCACGTCAACGTCAGTTACAACCTGCTCTCAACGCTTGCCATACCGATCG   1277

Ng_APL1A_1   CGGTCGAAGAGCTAGACGCATCGCACAATAGCATCAACGCCGTACGCGGGCCCGTCAACATGGAGTTGACCATTCTGAAGCTGCAACACAACAACTTGACTGACACTGCCTGGCTGGTGA   1144
Ng_APL1A_2   CGGTAAAAGAGCTACATGCATCGCACATTCGCATCAATGCCGTACGCGGGCCCTTCAACATGGAATTGACCATTCTGAAGCTCCAACACAACAACTTAACTGACACTGCCTGGCTGGTGA   1283
Ng_APL1A_3   CGGTCGAAGAGCTAGACGCATCGCACAATAGCATCAACGCCGTACGCGGGCCCGTCAACATGGAGTTGACCATTCTGAAGCTGCAACACAACAACTTGACTGACACTGCCTGGCTGTTGA   1144
Ng_APL1B_1   CAGTCGAAGAACTAGACGCATCGCACAATAGCATCAACGTCGTACGCGGGCCCGTCAACGTGGAGTTGACCATTCTGAAGCTGCAACACAACAACTTGACTGACACTGCCTGGCTGCTGA   939
Ng_APL1B_2   CGGTCGAAGAACTAGACGCATCGCACAATAGCATCAACGTCGTACGCGGGCCCGTCAACGTGGAGTTGACCATTCTGAAGCTGCAACACAACAACTTGACTGACACTGCCTGGCTGCTGA   939
Ng_APL1B_3   CAGTCGAAGAACTAGACGCATCGCACAATAGCATCAACGTCGTACGCGGGCCCGTCAACGTGGAGTTGACCATTCTGAAGCTGCAACACAACAACTTGACTGACACTGCCTGGCTGCTGA   939
Ng_APL1C_1   CGGTCGAAGAGCTAGACGCATCGCACAATAGCATCAACGCCGTACGCGGGCCCGTCAACGTGGAGTTGACCATTCTGAAGCTGCAACACAACAACTTGACTGACACTGCCTGGCTGCTGA   1427
Ng_APL1C_2   CGGTCGAAGAACTAGACGCATCGCACAATACCATCAACGTCGTACGCGGGCCCGTCAACGTGGAGTTGACCATTTTGAAGCTGCAACACAACAACTTGACTGACACTGCCTGGCTGCTGA   1427
Ng_APL1C_3   CGGTCGAAGAGCTAGACGCATCGCACAATAGCATCAACGCCGTACGCGGACCCGTCAACGTGGAGTTGACCATTCTGAAGCTGCAACACAACAACTTGACTGACACTGCCTGGCTGCTGA   1397
```

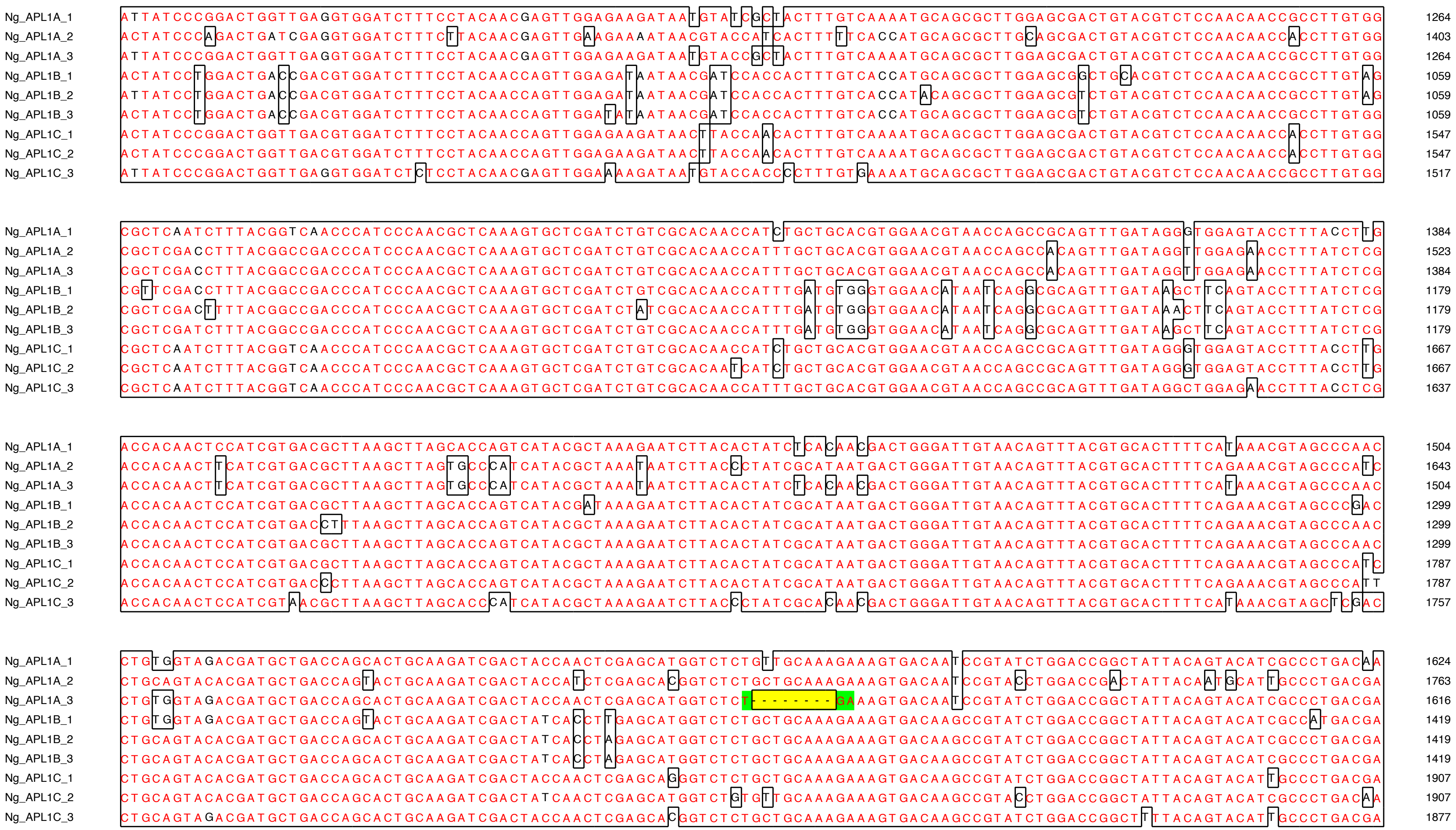

Ng_APL1A_1 1264
Ng_APL1A_2 1403
Ng_APL1A_3 1264
Ng_APL1B_1 1059
Ng_APL1B_2 1059
Ng_APL1B_3 1059
Ng_APL1C_1 1547
Ng_APL1C_2 1547
Ng_APL1C_3 1517
Ng_APL1A_1 1384
Ng_APL1A_2 1523
Ng_APL1A_3 1384
Ng_APL1B_1 1179
Ng_APL1B_2 1179
Ng_APL1B_3 1179
Ng_APL1C_1 1667
Ng_APL1C_2 1667
Ng_APL1C_3 1637
Ng_APL1A_1 1504
Ng_APL1A_2 1643
Ng_APL1A_3 1504
Ng_APL1B_1 1299
Ng_APL1B_2 1299
Ng_APL1B_3 1299
Ng_APL1C_1 1787
Ng_APL1C_2 1787
Ng_APL1C_3 1757
Ng_APL1A_1 1624
Ng_APL1A_2 1763
Ng_APL1A_3 1616
Ng_APL1B_1 1419
Ng_APL1B_2 1419
Ng_APL1B_3 1419
Ng_APL1C_1 1907
Ng_APL1C_2 1907
Ng_APL1C_3 1877

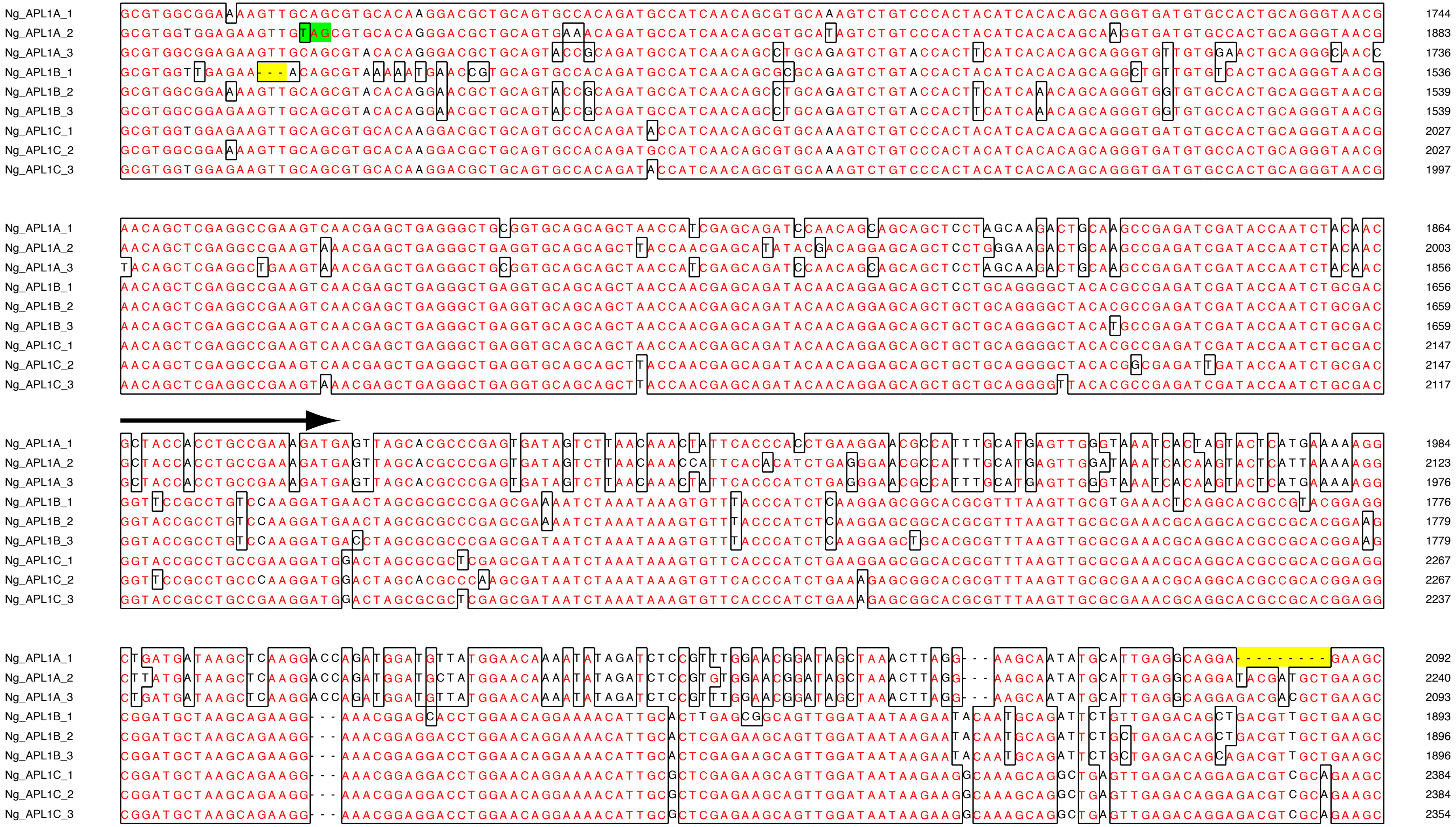
Ng_APL1A_1 1744
Ng_APL1A_2 1883
Ng_APL1A_3 1736
Ng_APL1B_1 1536
Ng_APL1B_2 1539
Ng_APL1B_3 1539
Ng_APL1C_1 2027
Ng_APL1C_2 2027
Ng_APL1C_3 1997
Ng_APL1A_1 1864
Ng_APL1A_2 2003
Ng_APL1A_3 1856
Ng_APL1B_1 1656
Ng_APL1B_2 1659
Ng_APL1B_3 1659
Ng_APL1C_1 2147
Ng_APL1C_2 2147
Ng_APL1C_3 2117
Ng_APL1A_1 1984
Ng_APL1A_2 2123
Ng_APL1A_3 1976
Ng_APL1B_1 1776
Ng_APL1B_2 1779
Ng_APL1B_3 1779
Ng_APL1C_1 2267
Ng_APL1C_2 2267
Ng_APL1C_3 2237
Ng_APL1A_1 2092
Ng_APL1A_2 2240
Ng_APL1A_3 2093
Ng_APL1B_1 1893
Ng_APL1B_2 1896
Ng_APL1B_3 1896
Ng_APL1C_1 2384
Ng_APL1C_2 2384
Ng_APL1C_3 2354

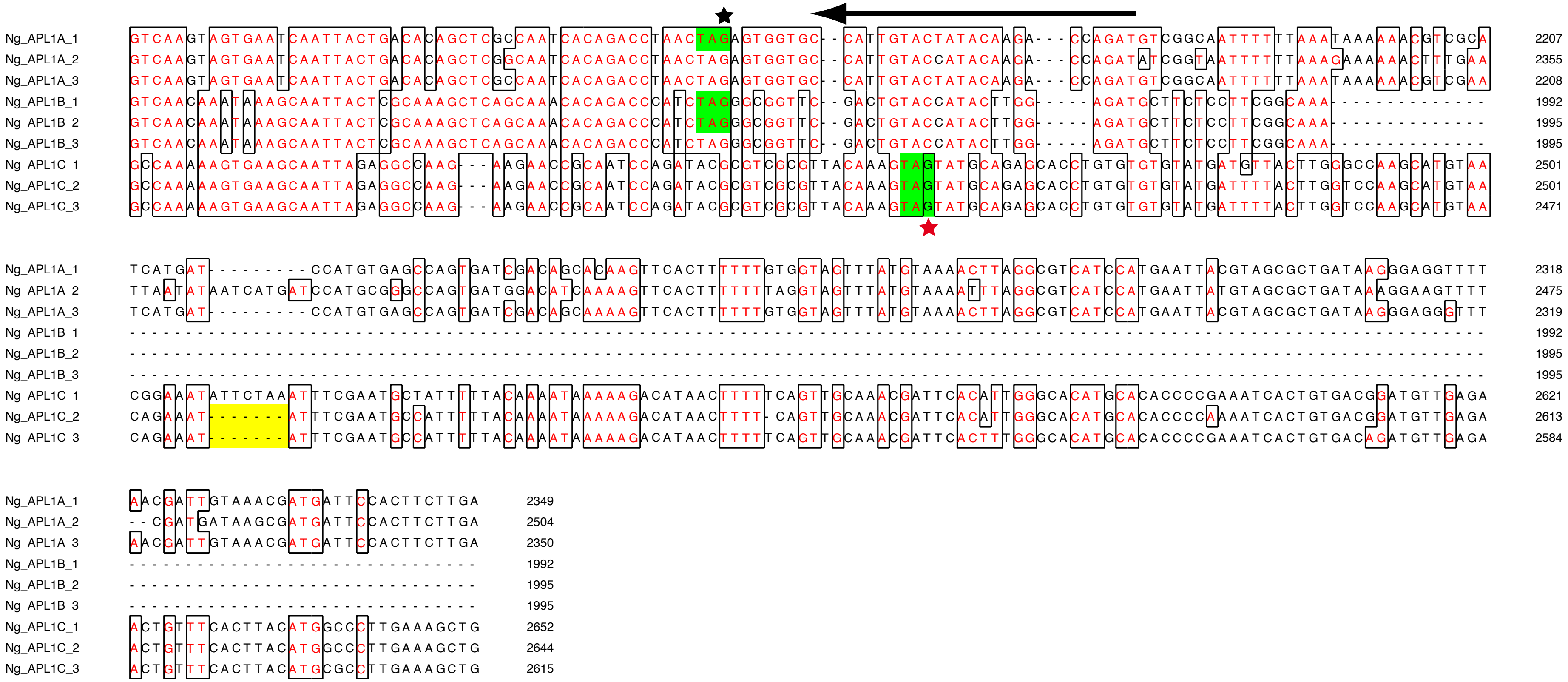
Ng_APL1A_1 2207
Ng_APL1A_2 2355
Ng_APL1A_3 2208
Ng_APL1B_1 1992
Ng_APL1B_2 1995
Ng_APL1B_3 1995
Ng_APL1C_1 2501
Ng_APL1C_2 2501
Ng_APL1C_3 2471
Ng_APL1A_1 2318
Ng_APL1A_2 2475
Ng_APL1A_3 2319
Ng_APL1B_1 1992
Ng_APL1B_2 1995
Ng_APL1B_3 1995
Ng_APL1C_1 2621
Ng_APL1C_2 2613
Ng_APL1C_3 2584
Ng_APL1A_1 2349
Ng_APL1A_2 2504
Ng_APL1A_3 2350
Ng_APL1B_1 1992
Ng_APL1B_2 1995
Ng_APL1B_3 1995
Ng_APL1C_1 2652
Ng_APL1C_2 2644
Ng_APL1C_3 2615
