# Supplementary figures and images for: Diverged Alleles of the Anopheles gambiae Leucine-Rich Repeat Gene APL1A Display Distinct Protective Profiles against Plasmodium falciparum
Source: PLoS One. 2012 Dec 28;7(12):e52684. doi: 10.1371/journal.pone.0052684 (PMC3532451; doi:10.1371/journal.pone.0052684)

**Figure S2: Observed infection intensities in silencing experiments**

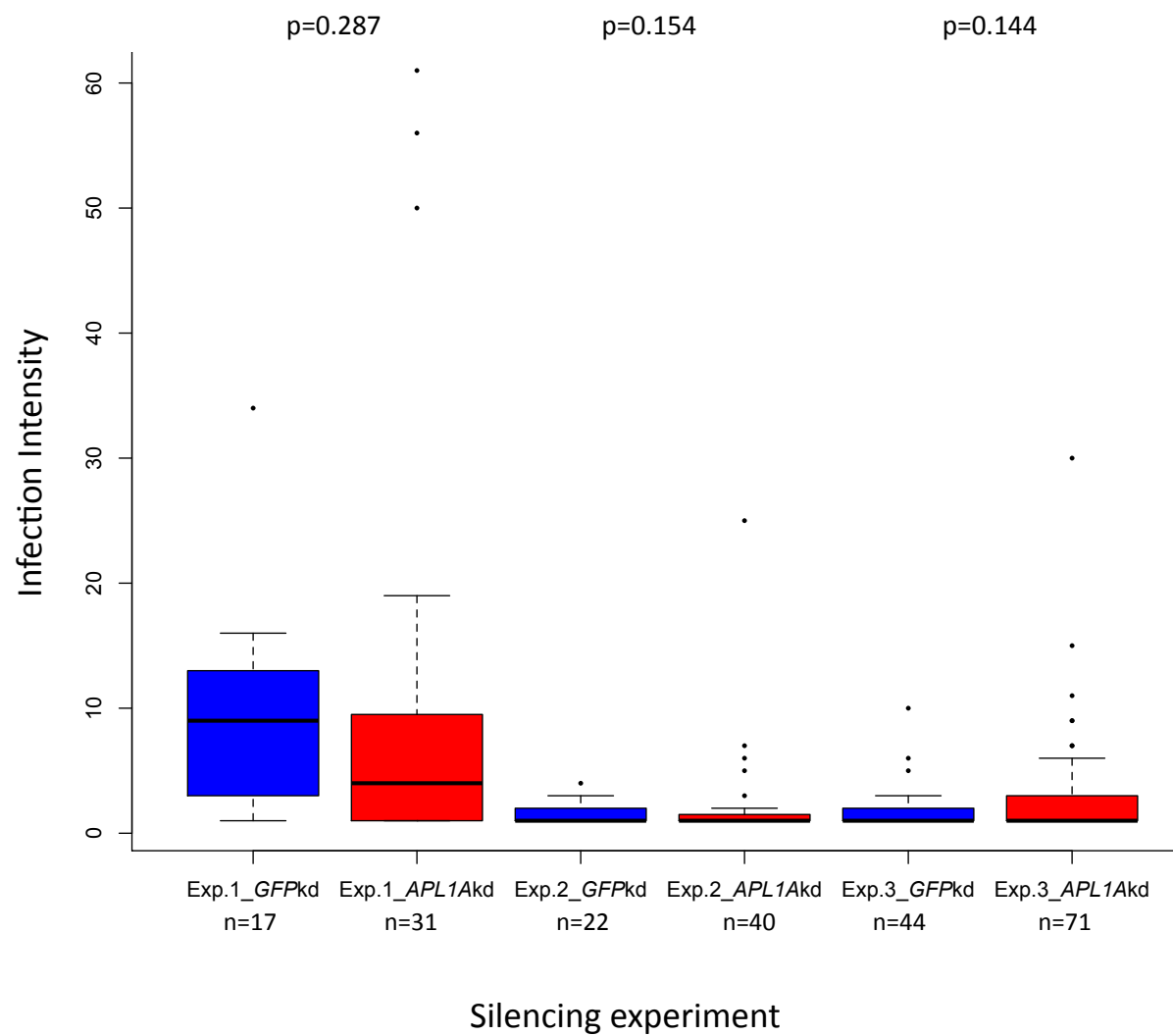

Supplement: Figure S2 — Observed infection intensities in silencing experiments. The experiment number (Exp.1, Exp.2 and Exp.3) and the RNAi knockdown target are shown on the horizontal axis. The number of infected mosquitoes (n) from each knockdown experiment is indicated. GFPkd was used as a control dsRNA. The vertical axis shows the number of midgut oocysts 7–8 days following a P. falciparum infectious blood meal. The median number of oocysts is indicated by the solid horizontal bar. The calculated p-values, comparing GFPkd with APL1Akd, indicate lack of a statistically significant effect of APL1A silencing on infection intensity in the three independent experiments. (PDF) [file pone.0052684.s002.pdf]

**Figure S3: Observed infection intensities without knockdown**

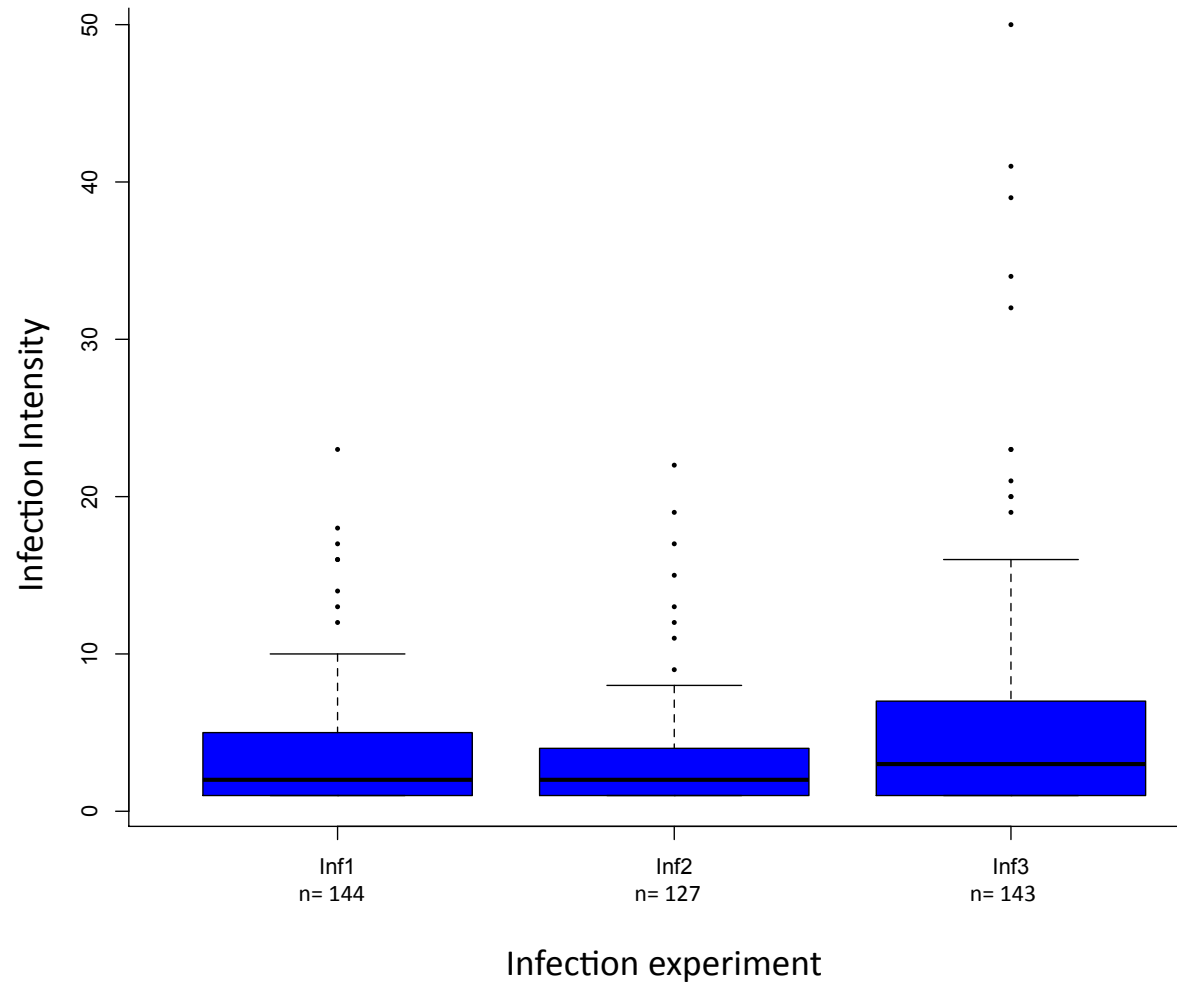

Supplement: Figure S3 — Observed infection intensities without knockdown. The experiment number (Inf1, Inf2 and Inf3) is shown on the horizontal axis. The number of infected mosquitoes (n) from each experiment is indicated. The vertical axis shows the number of midgut oocysts 7–8 days following a P. falciparum infectious blood meal. The median number of oocysts is indicated by the solid horizontal bar. (PDF) [file pone.0052684.s003.pdf]

**Figure S4A: Observed *APL1A* allele frequencies**

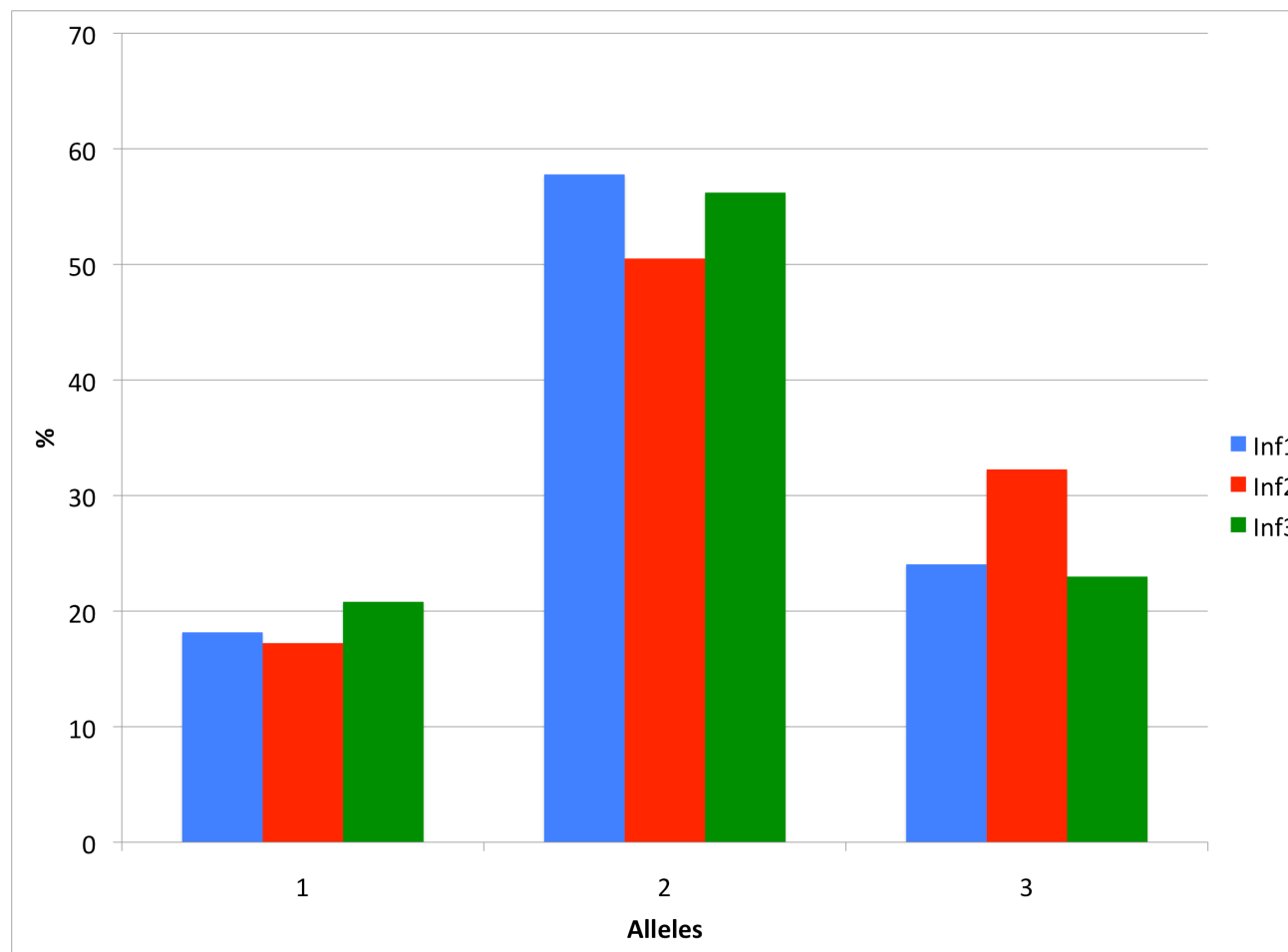

**Figure S4B: Observed *APL1A* genotype frequencies**

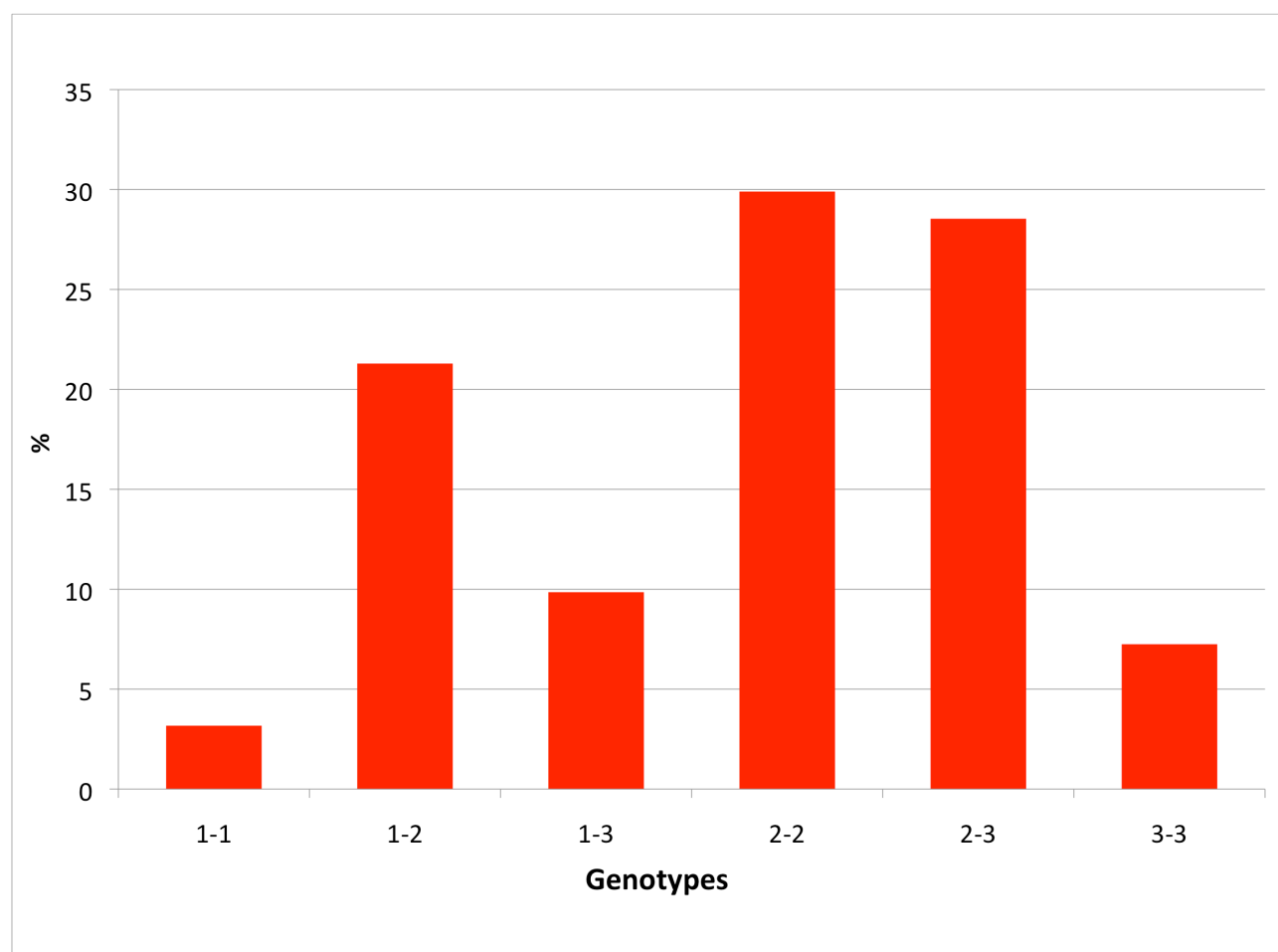

Supplement: Figure S4 — Determination of APL1A allele frequencies and genotypes in the Ngousso population with the APL1A -RFLP test. A) Observed APL1A allele frequencies. The histogram shows the allele composition (in percentage) of Ngousso females in three independent infection experiments (Inf1, Inf2 and Inf3). Numbers below the x-axis correspond to the alleles APL1A1 (1), APL1A2 (2) and APL1A3 (3), respectively. B) Observed APL1A genotype frequencies. The histogram shows the APL1A genotype composition (in percentage) of all Ngousso females from the three infection experiments (Inf1, Inf2 and Inf3) analyzed in figure S4A. Numbers below the x-axis correspond to following genotypes: APL1A1/APL1A1 (1-1), APL1A1/APL1A2 (1-2), APL1A1/APL1A3 (1-3), APL1A2/APL1A2 (2-2), APL1A3/APL1A2 (3-2) and APL1A3/APL1A3 (3-3). (PDF) [file pone.0052684.s004.pdf]
