# Supplementary material for: Diverged Alleles of the Anopheles gambiae Leucine-Rich Repeat Gene APL1A Display Distinct Protective Profiles against Plasmodium falciparum
Source: PLoS One. 2012 Dec 28;7(12):e52684. doi: 10.1371/journal.pone.0052684 (PMC3532451; doi:10.1371/journal.pone.0052684)
Supplement: Figure S5 — Polydot plot of APL1 genes and their allelic variants. Polydot software [2] has been used to perform pair wise comparisons of all Ngousso (Ng) APL1 alleles or their encoded proteins in order to illustrate their high degree of similarity. The word size used for the plots is indicated on top of the graphs. It corresponds to the length of the fragment that should have an exact match in both sequences used in the comparison. A: Polydot plot of APL1 alleles. For the comparison of all APL1 alleles in Ngousso, the corresponding genomic regions have been extracted, beginning at their start codon and up to the position of the stop codon of the longest allele of each gene, i.e.: APL1A (2L:41270938.41270940), APL1B (2L:41266619.41266621) and APL1C (2L:41257877.41257879). Red boxes highlight comparisons between alleles of the same gene (APL1A, APL1B of APL1C) and therefore show the alignments with the highest identity level. For APL1B and APL1C the nucleotide identity is high over the complete gene while for APL1A the differences between the alleles are visible: APL1A1 and APL1A3 display a nearly perfect diagonal while the APL1A2 allele harbors a different 5′end due to the repeat region which is similar to the APL1C alleles. It should be noted that the most divergent part on this plots between APL1A genes and APL1B/APL1C genes is the 3′ end where the dsRNA has been choosen (see Figure S1C for more details). B: Polydot plot of APL1 protein variants. The protein sequences of all APL1 variants were used in the comparison. To permit the comparison with the very short APL1B3 allele, its premature stop codon has been ignored, resulting in an artificial in silico product, which was extended to the same length as the other APL1B proteins. C: Polydot plot of APL1 genomic regions against the nucleotide sequence of the APL1A dsRNA regions. Oligonucleotides used for the synthesis of the dsAPL1A are positioned at (2L: 41271198.41271218) and downstream of the APL1A1 stop codon (2L:41270907.4 [file pone.0052684.s005.pdf]

Figure S5A: Polydot plot of *APL1* alleles

wordsize=8

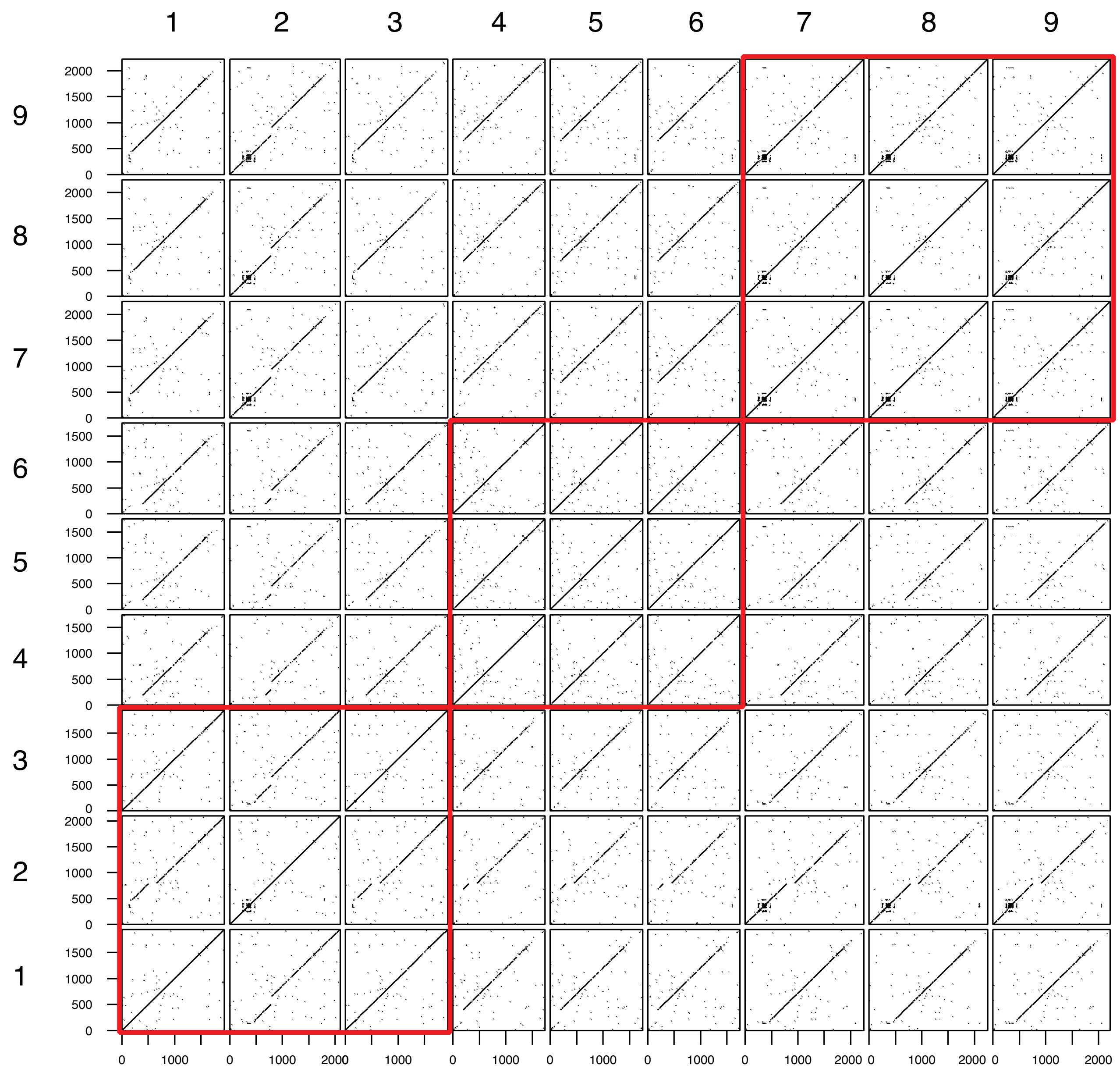

No	Sequence	Length
1	*Ng_APL1A_1*	1945
2	*Ng_APL1A_2*	2093
3	*Ng_APL1A_3*	1946
4	*Ng_APL1B_1*	1746
5	*Ng_APL1B_2*	1749
6	*Ng_APL1B_3*	1749
7	*Ng_APL1C_1*	2252
8	*Ng_APL1C_2*	2252
9	*Ng_APL1C_3*	2222

Figure S5B: Polydot plot of APL1 protein variants

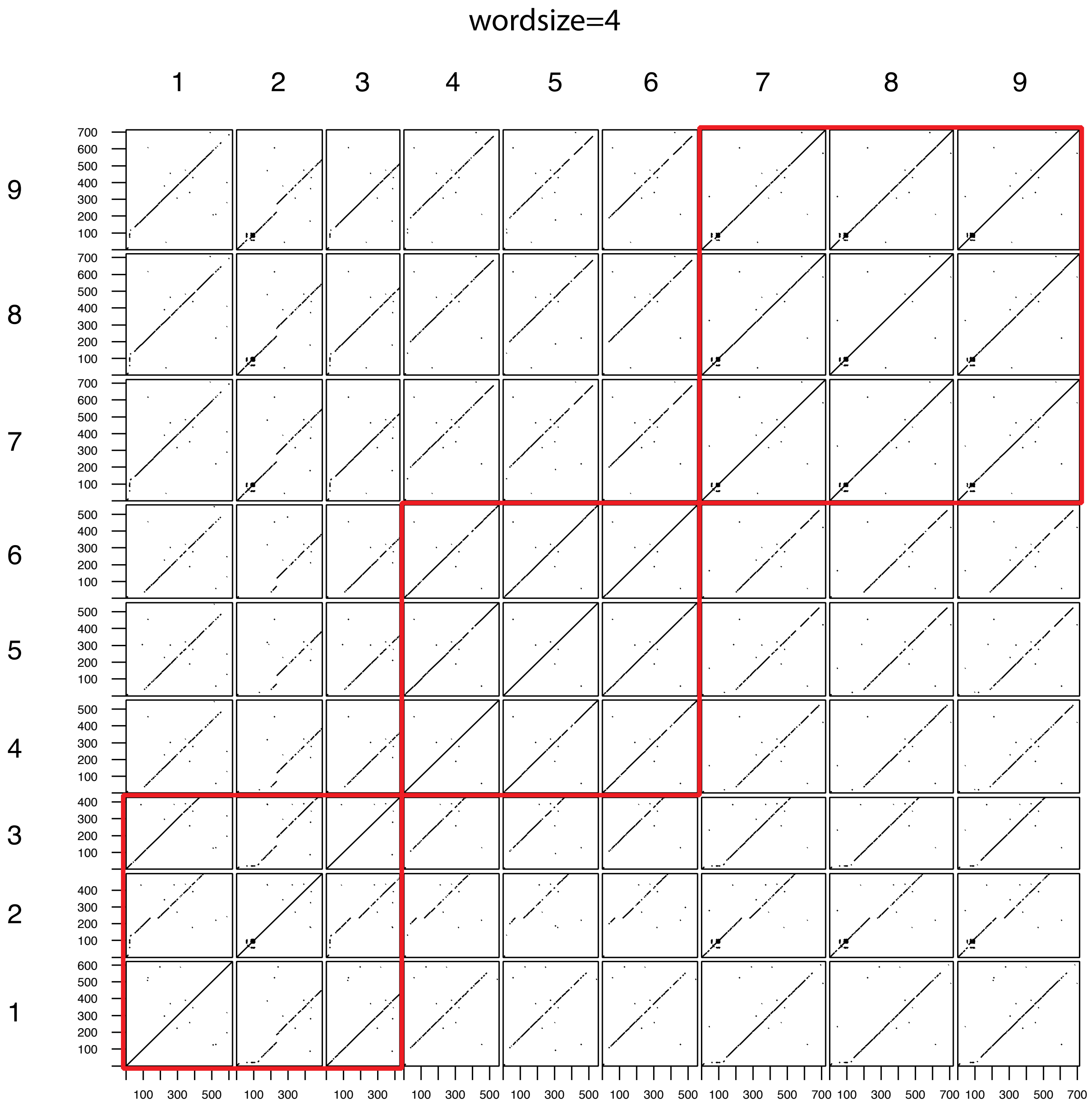

No	Sequence	Length
1	Ng_APL1A_1	621
2	Ng_APL1A_2	499
3	Ng_APL1A_3	428
4	Ng_APL1B_1	554
5	Ng_APL1B_2	555
6	Ng_APL1B_3	555
7	Ng_APL1C_1	722
8	Ng_APL1C_2	722
9	Ng_APL1C_3	712

Figure S5C: Polydot plot of *APL1* genomic regions against the nucleotide sequence of the *APL1A* dsRNA regions

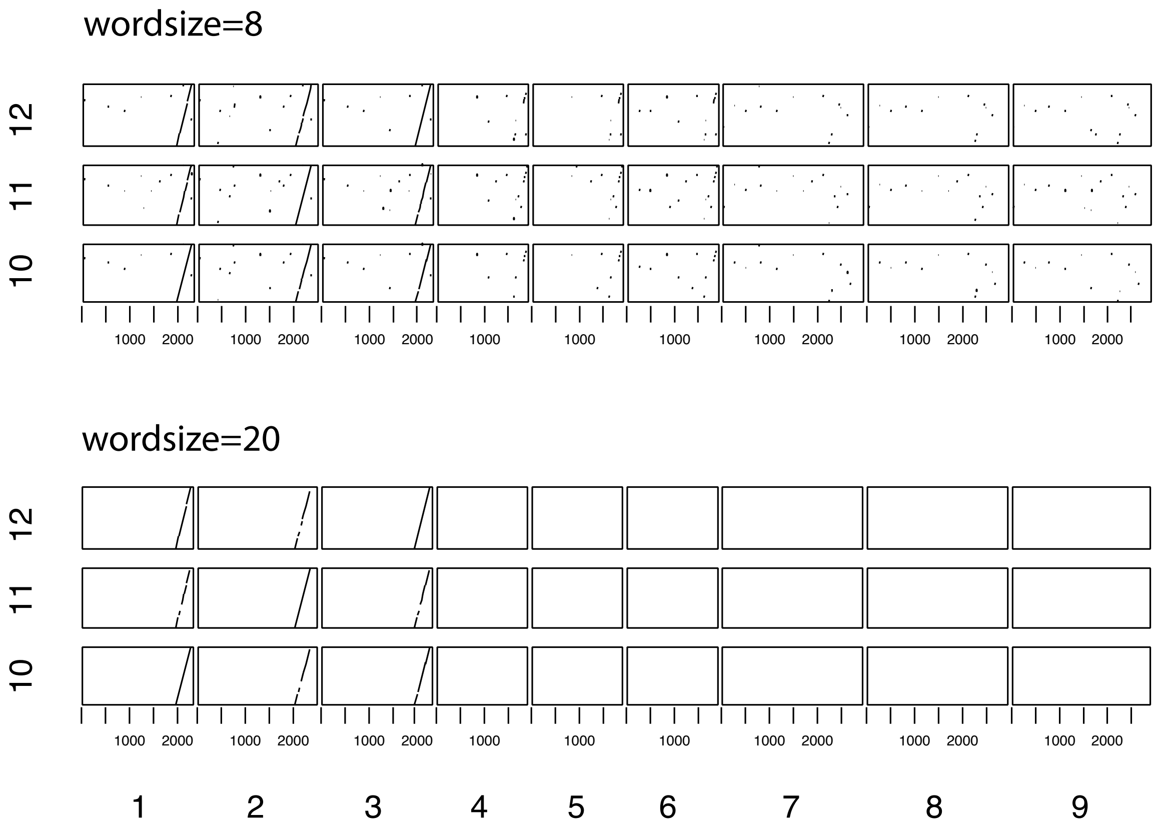

No.	Length	Sequence
1	2310	*Ng_APL1A_1*
2	2478	*Ng_APL1A_2*
3	2311	*Ng_APL1A_3*
4	1878	*Ng_APL1B_1*
5	1881	*Ng_APL1B_2*
6	1881	*Ng_APL1B_3*
7	2924	*Ng_APL1C_1*
8	2916	*Ng_APL1C_2*
9	2878	*Ng_APL1C_3*
10	312	dsNg_APL1A_1
11	321	dsNg_APL1A_2
12	321	dsNg_APL1A_3
