# Supplementary material for: Diverged Alleles of the Anopheles gambiae Leucine-Rich Repeat Gene APL1A Display Distinct Protective Profiles against Plasmodium falciparum
Source: PLoS One. 2012 Dec 28;7(12):e52684. doi: 10.1371/journal.pone.0052684 (PMC3532451; doi:10.1371/journal.pone.0052684)
Supplement: Table S1 — Oligonuclotides used to amplify APL1 genes and genomic regions from A. gambiae Ngousso. (PDF) [file pone.0052684.s006.pdf]

Table S1: Oligonuclotides used to amplify *APL1* genes and genomic regions from *A. gambiae* Ngousso.

genomic region	primer 1	primer 2
intergenic region: AGAP007037 - AGAP007036	5' CAAATCGCGCAAGATGATCAAGC 3'	5' TATTAGTGATACGGCGCGTAACC 3'
APL1A^{1} and *APL1A*^{3}	5' GATCTAAAGCCTAAGTCGACGTA 3'	5' TCGATCACTGGCTCACATGGAT 3'
APL1A^{2}	5' TAGCTGGATCCCAAGTAGTGCT 3'	5' CAGCGCTACGTAATTCATGG 3'
intergenic region: AGAP007036 - AGAP007035	5' CTCGGCAATCACAGACCTAACT 3'	5' CACCTACGGTAGAACTTGCTCA 3'
APL1B	5' TGAGCAAGTTCTACCGTAGGTG 3'	5' TTTGCCGAAGGAGAAGCATCTC 3'
APL1C	5' CCTCGAAAATCACACCAGACTG 3'	5' GAGCATCCCGTTTTCGATA 3'
