# Supplementary material for: Diverged Alleles of the Anopheles gambiae Leucine-Rich Repeat Gene APL1A Display Distinct Protective Profiles against Plasmodium falciparum
Source: PLoS One. 2012 Dec 28;7(12):e52684. doi: 10.1371/journal.pone.0052684 (PMC3532451; doi:10.1371/journal.pone.0052684)
Supplement: Table S2 — Parameters of the P. falciparum gametocyte (NF54) cultures and results of the different infection experiments. (PDF) [file pone.0052684.s007.pdf]

Table S2: Parameters of the *P. falciparum* gametocyte (NF54) cultures and results of the different infection experiments

A) Silencing experiments

	Exp.1	Exp.2	Exp.3
**Gametocytaemia**	1,4%	1,8%	2,0%
**Percentage of mature gametocytes**	83,50%	86,70%	94,10%

	Exp.1_*GFP*kd	Exp.1_*APL1A*kd	Exp.2_*GFP*kd	Exp.2_*APL1A*kd	Exp.3_*GFP*kd	Exp.3_*APL1A*kd
**Mosquito number (n)**	40	42	48	56	111	119
**Infection Prevalence (%)**	42	74	46	70	39	60
**Median Oocyst load**	9	4	1	1	1	1
**Mean Oocyst load**	9,2	10,1	1,7	2,1	1,7	2,8

B) Infection experiments without knock-down context

	Inf1	Inf2	Inf3
**Gametocytaemia**	1,70%	1,30%	2%
**Percentage of mature gametocytes**	90,60%	96,30%	94,10%

	Inf1	Inf2	Inf3
**Mosquito number (n)**	300	300	300
**Infection Prevalence (%)**	48	42	48
**Median Oocyst load**	2	2	3
**Mean Oocyst load**	3,8	3,2	5,9
