# Supplementary material for: Diverged Alleles of the Anopheles gambiae Leucine-Rich Repeat Gene APL1A Display Distinct Protective Profiles against Plasmodium falciparum
Source: PLoS One. 2012 Dec 28;7(12):e52684. doi: 10.1371/journal.pone.0052684 (PMC3532451; doi:10.1371/journal.pone.0052684)
Supplement: Supplementary Information S1 — (PDF) [file pone.0052684.s008.pdf]

SUPPLEMENTARY INFORMATION S1

Structure of *APL1* alleles in Ngousso

An insertion at the 5'end of the $APL1A^2$ variant encodes multiple repeats or variations of the peptide PANGGL, an amino acid repeat region normally only seen in APL1C. In addition, $APL1A^2$ has a 144 bp in-frame deletion that reduces the LRR domain by 48 aa as compared to the LRR domains found in the other two *APL1A* alleles.

At the 3'end, two small insertions, 8 and 9 bp in length, respectively, are diagnostic for *APL1A* alleles in Ngousso and neither is found in the PEST reference [1]. The 8bp insertion point is situated at position 2L:41271509-41271510 in the *APL1A* reference sequence and results in a frame-shift that suppresses the stop codon (2L:41271509-41271511). The insertion of 9 bp is situated at the very 3'end of the gene at position 2L:41270995-41270996. The $APL1A^1$ allele has only the 8 bp insertion, which extends the ORF to a stop codon at position 2L:41270938-41270940, leading to a protein with a C-terminal coiled-coil domain. The $APL1A^2$ allele, in addition to the N-terminal PANGGL repeat, has both, the 8 and 9 bp insertion. Here the 8 bp insertion is followed 69 bp further downstream (position 2L:41271439-41271441) by a premature stop codon due to a point mutation, resulting in an $APL1A^2$ protein lacking the C-terminal coiled-coil region. The $APL1A^3$ allele only displays the 9 bp insertion at the 3'end. This allele is similar to the *APL1A* allele of PEST, encoding a protein structured as the $APL1A^1$ form at its N-terminus but lacking the coiled-coil domain (Supplementary Figure 2A).

$APL1B^2$ *and* $APL1B^3$ variants have a 3 bp insertion (2L:41267132-41267133). The corresponding *APL1B* alleles form a haplotype with the *APL1A* variants that do not express the coiled-coil domain, $APL1A^2$ and $APL1A^3$, respectively.

In addition to the observed variations in the PANGGL encoding regions, the *APL1C* alleles of the *APL1* haplotypes 2 and 3 (variants $APL1C^2$ and $APL1C^3$, respectively) harbor a 7 bp deletion (at position 2L:41257813..41257819) compared to $APL1C^1$ and to the PEST reference genome sequence. This deletion, located 57 bp after the stop codon of *APL1C* (2L:41257877..41257879), does not change the gene product.

Homology of APL1 proteins

The three APL1A proteins display a high level of homology. The LRR regions, the common part of all three *APL1A* alleles, show 87% amino acid identity. Furthermore, the $APL1A^3$ protein is 94% identical to the $APL1A^1$ variant.

Concerning the *APL1B* products, if the premature stop codon in the *APL1B*^{3} allele is ignored and its coding sequence is virtually extended to the same length as the other *APL1B* variants, the three products share 93 to 97% amino acid identity in pair-wise comparisons. Similar results are found for the APL1C proteins. In addition, as illustrated by polydot graphs [2] the *APL1A*, *B* and *C* genes share extensive amino acid identity to each other (Figure S5).

Frequencies of *APL1A* alleles and genotypes in the Ngousso colony

Using the *APL1A*-RFLP typing assay, we determined allele and genotype frequencies in Ngousso mosquitoes from three independent infection series (with n=300 mosquitoes per replicate). The Ngousso colony displays different frequencies for the six *APL1A* genotypes, but despite variations in the genotype distributions, the frequencies of the three different *APL1A* alleles varied only slightly between the experiments (Figure S4). The three types of *APL1A* homozygotes are present in different frequencies in the Ngousso population with *APL1A*^{1}/*APL1A*^{1} homozygotes showing the lowest prevalence (Figure S4B). In all three experiments the genotypes were found to be in Hardy Weinberg equilibrium.

REFERENCES

1. Vectorbase http://www.vectorbase.org/.

2. EMBOSS http://emboss.sourceforge.net/.
